# Neratinib, a pan ERBB/HER inhibitor, restores sensitivity of *PTEN*-null, *BRAFV600E* melanoma to BRAF/MEK inhibition

**DOI:** 10.3389/fonc.2024.1191217

**Published:** 2024-05-16

**Authors:** Evan DuBose, Samantha M. Bevill, Dana K. Mitchell, Noah Sciaky, Brian T. Golitz, Shelley A. H. Dixon, Steven D. Rhodes, James E. Bear, Gary L. Johnson, Steven P. Angus

**Affiliations:** ^1^ Department of Pharmacology, University of North Carolina School of Medicine, Chapel Hill, NC, United States; ^2^ Department of Cell Biology and Physiology, University of North Carolina School of Medicine, Chapel Hill, NC, United States; ^3^ Department of Pediatrics, Herman B Wells Center for Pediatric Research, Indiana University School of Medicine, Indianapolis, IN, United States; ^4^ Indiana University Melvin and Bren Simon Comprehensive Cancer Center, Indianapolis, IN, United States; ^5^ Department of Medical and Molecular Genetics, Indiana University School of Medicine, Indianapolis, IN, United States; ^6^ Division of Pediatric Hematology/Oncology/Stem Cell Transplant, Indiana University School of Medicine, Indianapolis, IN, United States; ^7^ Lineberger Comprehensive Cancer Center, University of North Carolina School of Medicine, Chapel Hill, NC, United States; ^8^ Pharmacology & Toxicology, Indiana University School of Medicine, Indianapolis, IN, United States

**Keywords:** melanoma, kinase inhibitor, drug synergy, ERBB3, resistance

## Abstract

**Introduction:**

Approximately 50% of melanomas harbor an activating *BRAFV600E* mutation. Standard of care involves a combination of inhibitors targeting mutant BRAF and MEK1/2, the substrate for BRAF in the MAPK pathway. *PTEN* loss-of-function mutations occur in ~40% of BRAFV600E melanomas, resulting in increased PI3K/AKT activity that enhances resistance to BRAF/MEK combination inhibitor therapy.

**Methods:**

To compare the response of *PTEN* null to *PTEN* wild-type cells in an isogenic background, CRISPR/Cas9 was used to knock out *PTEN* in a melanoma cell line that harbors a *BRAFV600E* mutation. RNA sequencing, functional kinome analysis, and drug synergy screening were employed in the context of BRAF/MEK inhibition.

**Results:**

RNA sequencing and functional kinome analysis revealed that the loss of PTEN led to an induction of *FOXD3* and an increase in expression of the FOXD3 target gene, *ERBB3/HER3*. Inhibition of BRAF and MEK1/2 in *PTEN* null, *BRAFV600E* cells dramatically induced the expression of *ERBB3/HER3* relative to wild-type cells. A synergy screen of epigenetic modifiers and kinase inhibitors in combination with BRAFi/MEKi revealed that the pan ERBB/HER inhibitor, neratinib, could reverse the resistance observed in *PTEN* null, *BRAFV600E* cells.

**Conclusions:**

The findings indicate that *PTEN* null *BRAFV600E* melanoma exhibits increased reliance on ERBB/HER signaling when treated with clinically approved BRAFi/MEKi combinations. Future studies are warranted to test neratinib reversal of BRAFi/MEKi resistance in patient melanomas expressing ERBB3/HER3 in combination with its dimerization partner ERBB2/HER2.

## Introduction

While the genetic subset of mutations that contribute to melanoma are heterogeneous, ~50% of melanomas have an activating *BRAFV600E* mutation, which results in constitutive activation of the MEK1/2-ERK1/2 mitogen-activated protein kinase (MAPK) pathway (1). Aberrant ERK1/2 signaling leads to uncontrolled melanoma proliferation (2). The current clinical standard of care involves targeting BRAFV600E (or V600K) and MEK1/2, which elicits a potent antiproliferative response by inhibiting the ERK1/2 pathway (3–5). Despite improved response rates and extended progression-free survival with the combination of BRAFV600E and MEK1/2 inhibitors (BRAFi/MEKi), the development of resistance to these therapies and subsequent recurrence of disease remains a major clinical challenge (6, 7). Evaluation of factors influencing resistance identified loss-of-function mutations within the tumor-suppressor gene *PTEN* (phosphatase and tensin homolog deleted on chromosome 10), which occurs in 40% of sporadic melanomas and in up to 70% of melanoma cell lines (8, 9). *PTEN* is the second most commonly mutated tumor-suppressor gene in cancers, second only to *p53* (*TP53*), and is altered in various cancer types including glioblastoma, endometrial, breast, thyroid, and prostate cancers (10, 11). PTEN lipid phosphatase activity acts as a negative regulator of PI3K signaling by dephosphorylating PIP3 (phosphatidylinositol 3,4,5 tri-phosphate) to produce PIP2 (phosphatidylinositol 4,5 bi-phosphate). Functional PTEN loss activates PI3K/AKT signaling, which is a critical mediator of growth, survival, and apoptosis (12). *Braf* mutation in cooperation with *Pten* loss facilitates tumor formation and metastasis to distal organs in murine models of melanoma (13). PI3K pathway activation *via* loss of *PTEN*, which can suppress BIM-mediated apoptosis, or concurrent loss of *PTEN* and *RB1*, leads to BRAFi resistance in preclinical melanoma models (14–16). Clinically, patients with *PTEN* loss experience shorter progression-free survival when treated with the BRAFi, dabrafenib (17). Functional PTEN inactivation also confers resistance to anti-PD-1 immunotherapy in a preclinical model of melanoma due to increased expression of immunosuppressive cytokines in response to PI3K-AKT activation (18).

Here, we investigated the consequence of *PTEN* loss on BRAFi/MEKi resistance using RNA-seq and a chemical proteomics method (MIB/MS) to interrogate the functional kinome (19). The forkhead box D3 (FOXD3) transcription factor and its target gene *ERBB3* (*HER3*), a pseudokinase of the ERBB/HER receptor tyrosine kinase family, were induced in *PTEN* null *BRAFV600E* melanoma. ERBB3 (HER3) is a member of the epidermal growth factor receptor (EGFR) family that includes EGFR, ERBB2 (HER2), and ERBB4 (HER4). ERBB3 (HER3) is activated by neuregulin-1 or neuregulin-2, but since it lacks kinase activity, heterodimerization with another family member is required for downstream signaling. ERBB3/HER3 can directly bind to the regulatory p85 subunit of PI3Ks and drive PI3K/AKT signaling. RNA sequencing evidence of a potential FOXD3-ERBB3/HER3 signaling axis functioning as an adaptive regulator to BRAFV600E/MEK1/2 inhibition led us to explore the kinase response of *BRAFV600E*, *PTEN* null A375 melanoma cells. MIB/MS profiling of the global kinome revealed increased inhibitor bead capture of ERBB3/HER3 and AKT1/AKT3, indicating functional ERBB3/HER3-PI3K/AKT pathway activation. Drug synergy screens identified synergy between inhibitors targeting ERBB/HER kinases and BRAFi/MEKi. Treatment of *PTEN* null melanoma cells with BRAFi/MEKi therapy and the pan-ERBB/HER inhibitor, neratinib, attenuated growth when compared with either treatment alone. These results suggest that *BRAF* mutant, *PTEN*-deficient melanomas can adaptively shift to ERBB/HER dependence in response to BRAFi/MEKi and provide critical insight into the potential treatment of intrinsic or acquired resistance due to *PTEN* loss.

## Materials and methods

### Cell culture

The human melanoma cell lines used, A375 and SK-MEL-181, and the HEK293T line were obtained from Lineberger Comprehensive Cancer Center that were authenticated by short-tandem repeat (STR) testing. Cell lines were routinely tested by DAPI staining for mycoplasma. Cell lines were passaged no longer than 4–6 weeks for all experiments performed. Cells were cultured in DMEM (A375, HEK293T) or RPMI (SK-MEL-181), supplemented with 5% FBS and 1% penicillin–streptomycin.

### RNA interference

Short hairpin RNA constructs were purchased from Addgene. The pLKO.1-PTEN-shRNA-1320 plasmid (plasmid #25638, gift from Todd Waldman) was used to downregulate PTEN expression. The pLKO.1 puro scramble plasmid (plasmid #1864, gift from David Sabatini) was used as a non-targeting control. Transfection of pLKO.1 plasmids, psPAX2 (plasmid #12260, gift from Didier Trono), and pMD2.G (plasmid #12259, gift from Didier Trono) into HEK293T cells was performed using jetPRIME transfection reagent according to the manufacturer’s instructions (89129-922, Polyplus-transfection). Supernatant was collected 48 h after transfection, syringe-filtered (0.45 μm), and used for transduction of target cells in the presence of 8 μg/ml polybrene for 48 h before puromycin selection and maintenance (2.5 μg/ml) for 1 week prior to experiments.

### PI3K-overexpressing cell lines

The pLP-LNCX-PIK3CA-WT (plasmid #25633) and pLP-LNCX-PIK3CA-H1047R (Plasmid #25635, gifts from Todd Waldman) plasmids were purchased from Addgene. Plasmids were transfected into PLAT-A cells (20). The supernatant was collected 48 h later, filtered through 45 μm, and used to infect A375 cells (with 8 μg/ml polybrene). Two days later, polyclonal populations were selected and maintained in medium with 250 μg/ml G418.

### Immunoblotting

Melanoma cell lines were lysed in ice-cold multiplexed inhibitor bead (MIB) lysis buffer (50 mmol/L HEPES, 150 mmol/L NaCl, 0.5% Triton X-100, 1 mmol/L EDTA, 1 mmol/L EGTA, pH 7.5) supplemented with complete protease inhibitor cocktail (Roche) and 1% phosphatase inhibitor cocktails 2 and 3 (Sigma), and filtered lysates were boiled in 1× SDS sample buffer for 10 min at 100°C. Equal amounts of lysate were separated by SDS-PAGE, transferred to nitrocellulose membrane, blocked with 5% milk in TBS, and subjected to immunoblotting with the following primary antibodies: PTEN, phospho-ERK1/2 (218/222), phospho-AKT (S473), phospho-AKT (T308), AKT, HER2, FOXD3, and HER3 from Cell Signaling Technology; SOX10 and ERK2 from Santa Cruz Biotechnology. Secondary antibodies used were goat anti-rabbit-HRP or goat anti-mouse-HRP from Pierce Biotechnology. SuperSignal West Pico Chemiluminescent Substrate (Thermo Scientific) was used, and images were collected on a Bio-Rad ChemiDoc. Immunoblotting was performed at least three times, and representative images are shown.

### CRISPR/Cas9-mediated deletion of *PTEN*

CRISPR/PTEN KO plasmid (sc-400103) and PTEN HDR plasmid (sc-400103-HDR) pools were purchased from Santa Cruz. 2 μg of each plasmid was transfected into A375 cells using jetPRIME reagent, and cells were selected with 2.5 μg/ml puromycin for 1 week. Sanger sequencing identified three unique sgRNA sequences in the pool targeting human *PTEN* (cctacctctgcaattaaatt, ttatccaaacattattgcta, and accgccaaatttaattgcag). Single-cell clones were isolated by limiting dilution and screened by western blotting for loss of detectable PTEN expression. *PTEN* KO clones and the parental A375 line were then infected with Ad-Cre-GFP adenovirus (approximate MOI 30, resulting in ~95%–99% GFP positive cells) from Vector Biolabs (Cat. No. 1700).

### Immunofluorescent cell staining

Cells were fixed with 4% paraformaldehyde in Krebs S-buffer for 10 min and permeabilized in 0.2% Triton X-100 in PBS for 5 min at room temperature. Cells were blocked for 30 min in PBS containing 5% BSA. The primary antibody, diluted to 1:100 in PBS containing 5% BSA, was applied for 3 h at ambient temperature. This was followed by extensive washes in PBS. The fluorescent dye–conjugated secondary antibody and phalloidin dye were diluted to 1:400 in 5% BSA in PBS and applied for 1 h at ambient temperature followed by extensive washes in PBS. Cells were stained with 0.2 μg/ml Hoechst 33342 for 10 min at room temperature. The dye was washed out and Fluoromount-G (Electron Microscopy Sciences) was used as the mounting medium for fixed cells on coverslips. Fluorescent images were acquired on a Zeiss LSM800 confocal microscope using a 63× objective. The commercial antibody used for immunohistochemistry was purchased from Cell Signaling Technology (rabbit anti-PTEN). Alexa Fluor 568 (goat anti-rabbit), Phalloidin (Alexa Fluor 488), and Hoechst 33342 were purchased from Thermo Fisher Scientific. Staining was performed once on three technical replicates.

### Dose–response curves

Dose–response curves were performed in 384-well plates. Cells were plated 1 day prior to first treatment using a BioTek microplate dispenser. The following day, cells were dosed with drug using a Beckman Coulter Biomek FX instrument. Cells were treated with 0.001 nM–100 nM dabrafenib in half log doses and 0.0001 nM–10 nM trametinib in half log doses and in combination. For screening validation, triple-combination dose–response curves were performed by adding 100 nM neratinib to dabrafenib/trametinib combination treatment. DMSO was used as a negative control for growth inhibition on each plate. Plates were incubated at 37°C for 96 h and lysed by adding 10 μl CellTiter-Glo reagent (Promega, Cat. No. G7570) to 50-μl cell media. Luminescence was measured using a PHERAstar FS instrument, and growth inhibition was calculated relative to DMSO-treated wells. Dose–response curves were performed three times, with six technical replicates at each dose. Representative results from a single experiment are shown.

### Cell line growth assays

The growth assays were performed in 96-well plates. Cells were plated 1 day prior to first treatment. For multiday treatments, media containing fresh drug were changed every 3 days unless otherwise noted. Live cells were stained with Hoechst 33342 at 1:10,000 in PBS for 20 min at 37°C and imaged/counted with a Thermo Cellomics ArrayScan VTI at 12 frames per well. Growth assays were performed using six technical replicates and at least twice, with representative data shown from a single experiment.

### Crystal violet colony formation assays

Crystal violet assays were performed in six-well plates, with three technical replicates per condition. Cells were plated at a low density (2,000 cells per well) 1 day prior to first treatment. After 7 days, cells were rinsed with PBS, fixed in methanol (10 min, −20°C), and stained with 0.5% crystal violet for 20 min. Images were collected, and then crystal violet was solubilized with 30% acetic acid and quantified by absorbance at 600 nm. Crystal violet colony formation assays were performed at least three separate times and representative data are shown.

### Senescence-associated beta-galactosidase staining

Cells were seeded in six-well dishes 1 day prior to drug treatment. After a 96-h treatment with DMSO, 100 nM neratinib, 10 nM dabrafenib, and 1 nM trametinib, or the combination of dab/tram and neratinib, cells were rinsed in PBS and then fixed and processed for senescence-associated (SA)-beta-galactosidase staining according to the manufacturer’s protocol (Senescence Beta-Galactosidase Staining Kit, Cell Signaling Technology, Cat. No. 9860). Nuclei were counterstained with Hoechst 33342 prior to imaging. Experiments were performed three times, and representative data are shown.

### 3D spheroid assay

A375 wild-type, PTEN KO5, and PTEN KO11 cells were plated in low-adhesion round-bottom 96-well plates in biological triplicate. For spheroids, 5,000 cells were plated per well. Spheroids were given 72 h to form before beginning drug treatment. Before beginning drug treatment and at end point (7 days), brightfield images were captured using a Celigo instrument. Following imaging, spheroids were lysed in CellTiter-Glo 3D (Promega, Cat. No. G9681) by adding 50 μl reagent to 150-μl cell media. Plates were rotated at 150 rpm for 30 min before luminescence was read using a PHERAstar FS.

### Compounds

Information on compounds used in screening and subsequent validation experiments is listed in **Supplementary Table S4**. Dabrafenib, trametinib, vemurafenib, cobimetinib, encorafenib, and binimetinib were purchased from Selleck and dissolved in DMSO. Cultures were treated with 0.1% DMSO as control.

### Epigenetic/kinase inhibitor synergy screening

The optimal dose range for dabrafenib and trametinib was determined for each cell line screened across half log doses. Dabrafenib/trametinib screens were performed in 6 × 6 dose–response matrices. A375 wild-type, KO5, and KO11 cell lines were treated with 3 nM–1 μM dabrafenib/trametinib in half log doses. Cells were seeded in 384-well plates using a BioTek microplate dispenser. The following day, cells were dosed with drug using a Beckman Coulter Biomek FX instrument. The screening library was tested for growth inhibition alone or in combination across six doses of the screening library: 0.1 nM, 1 nM, 10 nM, 100 nM, 1 μM, and 10 μM. Bortezomib (1 μM) was included as a positive control or 0.1% DMSO as a negative control for growth inhibition on each plate. Plates were incubated at 37°C for 96 h and lysed by adding 10 μl CellTiter-Glo reagent (Promega, Cat. No. G7570) to 50-μl cell media. Luminescence was measured using a PHERAstar FS instrument, and growth inhibition was calculated relative to DMSO-treated wells. The full drug library synergy screen was performed once, and selected compounds and combinations were used for validation as indicated.

### Drug synergy analysis

Drug synergy scores were generated using the SynergyFinder package 1.6.1^33^. Bliss scores were calculated without baseline correction and using default parameters with the exception that Emin was specified as 0 and Emax as 100 (**Supplementary Table S5**). Synergy was assessed across individual doses of each library compound to generate six possible scores per compound. To be considered a hit, a given compound had to generate a positive mean synergy score for at least one dose tested in the PTEN KO cell lines screened. Hits were then ranked by the mean of all synergy scores produced in the drug combination matrix. Synergy scores represent the percent growth inhibition induced by a drug combination which exceeded the expected growth inhibition. Expected growth inhibition was calculated based on the effect of each drug as a single agent.

### RNA isolation, RNA-seq library preparation, sequencing, and analysis

PBS-washed A375 cell pellets were snap frozen on dry ice and sent to GENEWIZ Azenta Life Sciences (South Plainfield, NJ) for mRNA sequencing in biological triplicate. RNA was isolated from snap-frozen cell pellets using the RNeasy Plus Mini Kit (Qiagen) according to the manufacturer’s protocol with the optional DNase I treatment (Qiagen) for 15 min. Libraries were prepared using the NEBNext Ultra II RNA Library Prep with PolyA selection, 500 ng input, 10 PCR amplification protocols. Paired-end sequencing (2 × 150 bp) of equimolar amounts of each library was performed on an Illumina HiSeq 4000. Sequence reads were trimmed to remove possible adapter sequences and nucleotides with poor quality using Trimmomatic v.0.36 w (average 23.5 × 10^6^ reads per sample, mean quality score 35.2, mean 92.5% reads Q ≥30). The trimmed reads were mapped to the Homo sapiens GRCh38 reference genome available on ENSEMBL using the STAR aligner v.2.5.2b. The STAR aligner is a splice aligner that detects splice junctions and incorporates them to help align the entire read sequences. BAM files were generated as a result of this step. After alignment to GRCh38, mean 97.4% reads were successfully aligned. Unique gene hit counts were calculated by using featureCounts from the Subread package v.1.5.2. The hit counts were summarized and reported using the gene_id feature in the annotation file. Only unique reads that fell within exon regions were counted. After extraction of gene hit counts, the gene hit counts table was used for downstream differential expression analysis. Using DESeq2 (21), a comparison of gene expression between defined groups of samples was performed. The Wald test was used to generate p-values and log2 fold changes. Genes with an adjusted p-value < 0.05 (BH method) and absolute log2 fold change > 1 were called as differentially expressed genes for each comparison and displayed as volcano plots. DESeq2 results for pairwise comparisons are provided as **Supplementary Table S3**.

Raw counts were imported into iDEPv0.96 (22) and preprocessed using default parameters, and normalized counts (**Supplementary Table S1**) were used for unsupervised hierarchical clustering and k-means clustering using default parameters (top 2,000 genes, Pearson correlation, average linkage for hierarchical clustering and top 2,000 genes, four clusters for K-means clustering). K-means clustering heatmap data are provided as **Supplementary Table S2**.

### TCGA skin cutaneous melanoma expression analysis

From cbioportal.org, we identified cutaneous melanoma samples in the TCGA PANCAN data set harboring *BRAFV600E* or *BRAFV600K* mutations (23, 24). Within this data set, we compared the expression of *SOX10, FOXD3, ERBB2*, and *ERBB3* in samples with *PTEN* alterations (driver mutations (missense, nonsense, frameshift) or predicted homozygous deletion), n = 43, to those without, n = 143, using expression data obtained from xenabrowser.net (25). Samples with nulls were excluded. Outliers were removed using GraphPad Prism Robust regression and outlier removal (ROUT) method (Q = 1%). Statistical analysis between groups was performed using two-tailed unpaired Student’s t test. *P* < 0.05 was considered significant.

Outliers:

**Table:** 

	*PTEN* wt	*PTEN* mut
*SOX10*	2	0
*FOXD3*	3	1
*ERBB2*	1	0
*ERBB3*	4	0

### Multiplexed inhibitor bead chromatography and mass spectrometry (MIB/MS) analysis

MIB/MS experiments were performed as described previously (19, 26). Briefly, snap-frozen melanoma cells were lysed in ice-cold MIB lysis buffer (150 mmol/L NaCl). Extracts were sonicated 3 × 10 seconds, clarified by centrifugation, and syringe-filtered (0.22 μm) prior to Bradford assay quantitation of concentration. Equal amounts of total protein (0.3 mg) were gravity-flowed over MIB columns in high salt MIB lysis (1 mol/L NaCl). Bound protein was eluted twice with 0.5% SDS, 1% β-mercaptoethanol, 100 mmol/L Tris–HCl, pH 6.8 for 15 min at 100°C. Eluate was treated with DTT (5 mmol/L) for 25 min at 60°C and 20 mmol/L iodoacetamide for 30 min in the dark. Following spin concentration using Amicon Ultra-4 (10k cutoff) to approximately 100 μL, samples were precipitated by methanol/chloroform, dried in a SpeedVac, and resuspended in 50 mmol/L HEPES (pH 8.0). Tryptic digests were performed overnight at 37°C and peptides further cleaned using C-18 spin columns according to the manufacturer’s protocol (Pierce). Peptides were resuspended in 2% ACN and 0.1% formic acid. 40% of the final peptide suspension was injected onto a Thermo EASY-Spray 75 μm × 25 cm C-18 column and separated on a 180-min gradient (5%–40% ACN) using an EASY nLC-1000. Raw files were processed for label-free quantification (LFQ) by MaxQuant LFQ using the UniProt/Swiss-Prot human database. Kinases with fewer than 2 razor+unique peptides were excluded. Normalized LFQ intensities were imported into Perseus software. In Perseus, LFQ intensities were log_2_-transformed and missing values were imputed by column using default parameters for each sample to enable comparison (**Supplementary Table S6**).

### Data availability

The RNA-seq datasets generated during and/or analyzed during the current study are available in the Gene Expression Omnibus (GEO) (Accession no. GSE255541). The mass spectrometry proteomics data have been deposited to the ProteomeXchange Consortium *via* the PRIDE partner repository with the dataset identifier PXD033866 (Username: reviewer_pxd033866@ebi.ac.uk, Password: qst3pRLr) and PXD033921 (Username: reviewer_pxd033921@ebi.ac.uk, Password: 02pkX3Zp). All other data generated and/or analyzed during this study are included in this article (and its **Supplementary Information Files**).

## Results

### *PTEN* loss in *BRAFV600E* melanoma cells decreases sensitivity to dabrafenib/trametinib-induced growth arrest

**Figure 1:**
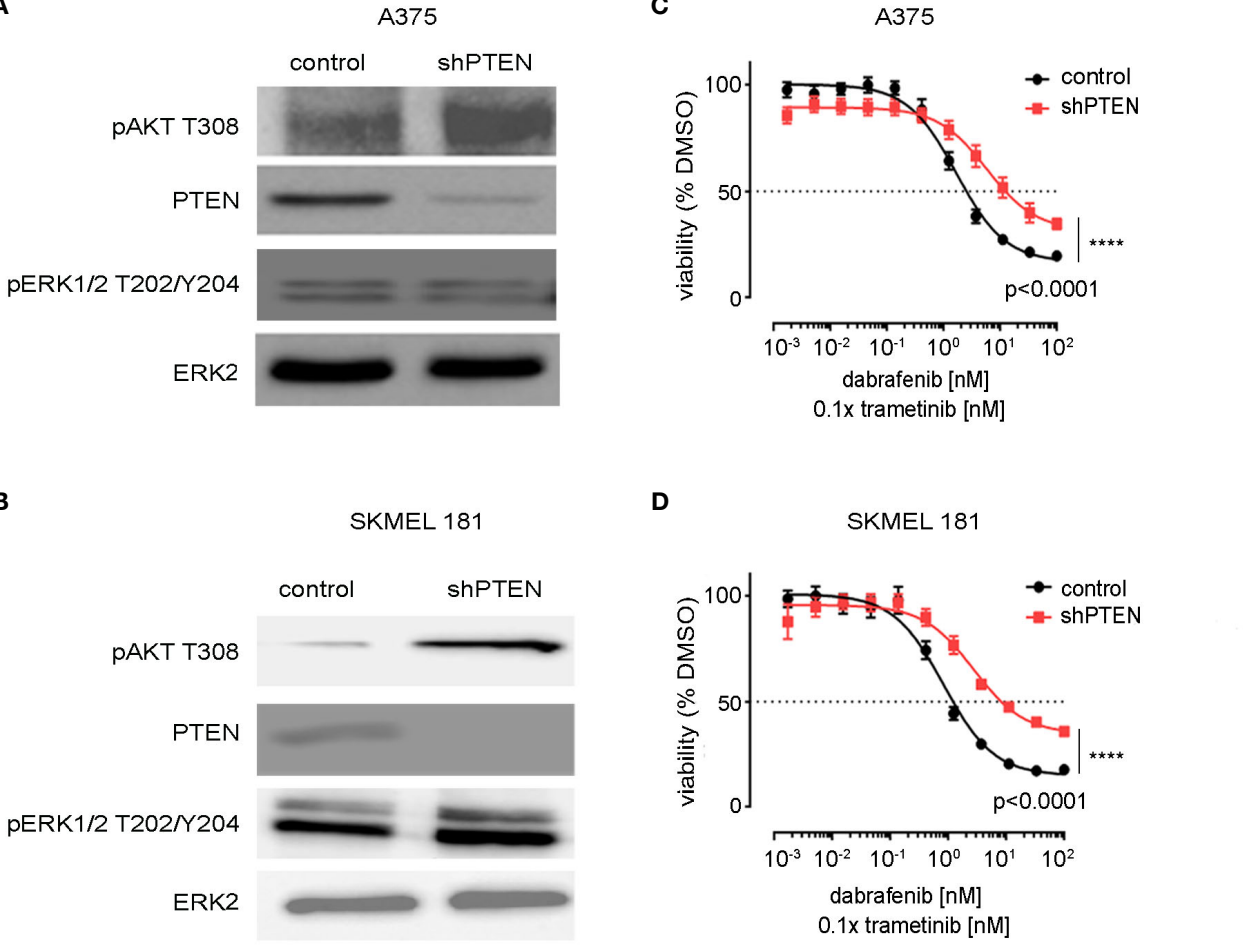
Knockdown of PTEN in BRAFV600E melanoma cell lines. **(A)** A375 and **(B)** SK-MEL-181 cells expressing non-targeting (scrambled control) shRNA or shRNA targeting PTEN were generated by lentiviral infection and puromycin selection. Polyclonal populations were harvested and immunoblotting performed using the indicated antibodies. ERK2 was used as a loading control. Full-length blots are presented in **Supplementary Figure S9**. **(C)** A375 and **(D)** SK-MEL-181 cells were treated with DMSO (control) or the indicated dose of dabrafenib/trametinib for 96 h prior to viability measurement using CellTiter-Glo. Dabrafenib (1×) and trametinib (0.1×) were used at 10:1 dose ratio. Mean +/− SD, n = 6. Indicated p-values were calculated using the extra sum of squares F-test in Prism.

*PTEN* expression was inhibited by shRNA in A375 and SK-MEL-181 human melanoma cell lines, both of which express BRAFV600E and wild-type PTEN (27) (**Figure 1**). Immunoblotting confirmed decreased PTEN protein levels with shRNA knockdown in both the A375 and SK-MEL-181 cells as well as increased phosphorylation of AKT, consistent with predicted activation of the PI3K pathway (**Figures 1A, B**) (9). Treatment of both *PTEN* knockdown lines relative to control cells with increasing concentrations of dabrafenib (BRAFi) and trametinib (MEKi) showed a decrease in sensitivity to growth inhibition with PTEN loss compared with control (**Figures 1C, D**).

Activation of the PI3K-AKT pathway *via* mutations in the PI3Kα gene, *PIK3CA*, have also been observed in melanoma (28). To determine if activated PI3K would mimic *PTEN* loss in *BRAFV600E* melanoma, Flag-tagged wild-type PI3K (PI3KWT) and the activated *PIK3CA* H1047R mutant were ectopically expressed in A375 cells (**Supplementary Figure S1A**). The H1047R mutation increases catalytic activity of PI3K (29). Expression of *PIK3CA* H1047R decreased the sensitivity to dabrafenib/trametinib growth inhibition relative to expression of wild-type PIK3CA (**Supplementary Figures S1B, C**). The findings indicate PTEN loss, and activating PI3K mutations confer comparable resistance to BRAFi/MEKi.

**Figure 2:**
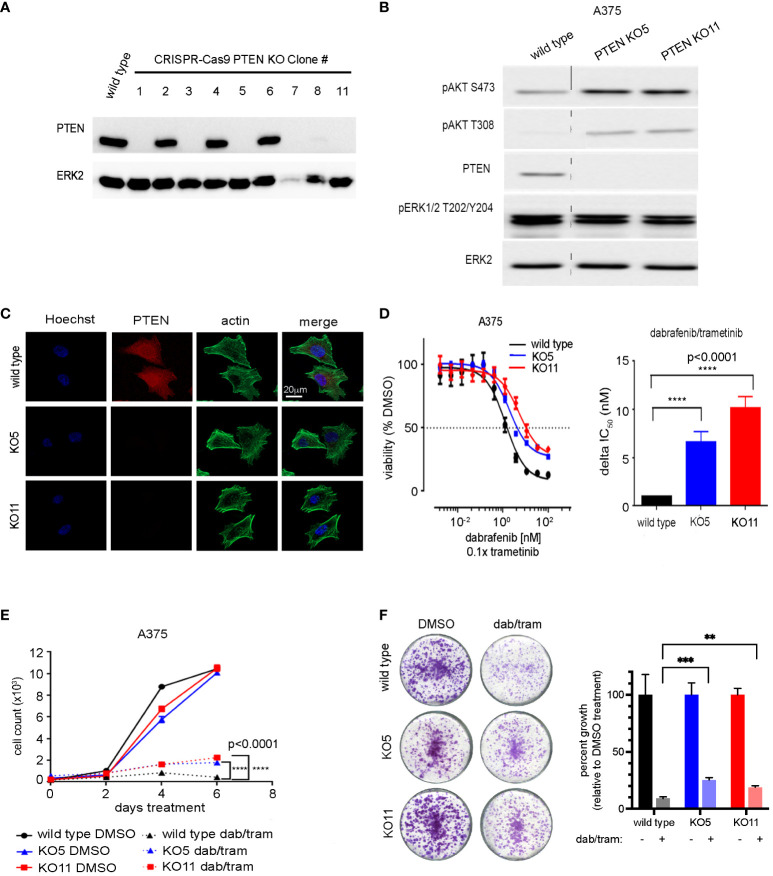
CRISPR-mediated PTEN loss decreases sensitivity of BRAFV600E melanoma cells to combined dabrafenib/trametinib inhibition. **(A)** A375 cells were transfected with a single-guide RNA pool targeting human PTEN and selected with puromycin. Single-cell clones were isolated, harvested, and immunoblotting was performed with the indicated antibodies. ERK2 was used as a loading control. Full-length blots are presented in **Supplementary Figure S8**. **(B)** A375 wild-type, PTEN null 5 (KO5), and PTEN null 11 (KO11) cell lines were harvested for immunoblotting with the indicated antibodies. ERK2 was used as a loading control. Full-length blots are presented in **Supplementary Figure S10**. **(C)** A375 wild-type, KO5, and KO11 cell lines were grown on glass cover slips, fixed, and immunofluorescent staining was performed for PTEN, actin (Phalloidin), and Hoechst 33342 (nuclear marker). Scale bar, 20 µm. **(D)** A375 wild-type, KO5, and KO11 cell lines were treated with DMSO (control) or the indicated dose of dabrafenib/trametinib for 96 h prior to viability measurement using CellTiter-Glo. Dabrafenib (1×) and trametinib (0.1×) were used at a 10:1 dose ratio. Graphical representation of a single experiment. Bar plot of delta IC_50_ values calculated by comparing fold change in IC_50_ in the wild-type vs. each PTEN KO cell line. Indicated p-values were calculated using two-tailed t-tests, n = 7. **(E)** Six-day cell growth curves across A375 wild-type, KO5, and KO11 cell lines treated with DMSO (control) or 10/1 nM dabrafenib/trametinib. Mean +/− SD, n = 6. Indicated p-values were calculated using two-tailed t-tests. **(F)** One-week crystal violet assays in wild-type, KO5, and KO11 cell lines. Cells were dosed with DMSO (control) or 10/1 nM dabrafenib/trametinib. Bar plot represents quantification of crystal violet stain, mean +/− SD, n = 3. Indicated p-values were calculated using two-tailed t-tests. *P < 0.05, **P < 0.01, ***P < 0.001, ****P < 0.0001.

To better model the effects of *PTEN* loss, CRISPR/Cas9 was employed to delete *PTEN* in the A375 melanoma cell line. Isogenic clones were isolated and their PTEN expression levels determined by immunoblotting (**Figure 2A**). As ERK2 levels appeared reduced in KO7, we confirmed that ERK2 was a suitable loading control and not an off-target of PTEN disruption by repeating the transfection and selection and utilizing additional loading controls, at baseline and in response to drug treatment (**Supplementary Figures S1A, B**). Two independent clones, *PTEN* null 5 and *PTEN* null 11 (KO5 and KO11) were chosen for further characterization based on their similar morphologies and growth rates to the wild-type A375 cells. Similar to the *PTEN* shRNA knockdown cells, *PTEN* null KO5 and KO11 cells had increased phospho-AKT with no change in phospho-ERK relative to parental wild-type cells (**Figure 2B**). Immunofluorescent staining of PTEN confirmed lack of expression in the PTEN null KO5 and KO11 cells with no overt morphological changes as a result of PTEN loss (**Figure 2C**).

Dose–response relationships of BRAFi/MEKi by dabrafenib/trametinib in *PTEN* null KO5 and KO11 and parental A375 cells were assessed to determine changes in drug sensitivity with loss of PTEN expression. Similar to *PTEN* shRNA knockdown cells, *PTEN* null cells showed a diminished sensitivity to growth inhibition with increasing concentrations of dabrafenib/trametinib compared with wild-type A375 cells. The half maximum inhibitory concentration (IC_50_) was analyzed over multiple experiments and demonstrated a highly reproducible, significant change in IC_50_ resulting from PTEN loss. The disruption of *PTEN* resulted in a 6.5-fold and 10-fold increase in KO5 and KO11 IC_50_, respectively, relative to the wild-type cells (**Figure 2D**). A similar shift in sensitivity to growth inhibition was seen with additional FDA-approved BRAFi/MEKi drug combinations, including encorafenib/binimetinib and vemurafenib/cobimetinib (30, 31), consistent with the observed resistance being a function of PTEN loss and not the specific BRAFi/MEKi combination (**Supplementary Figures S3A, B**).

Short- and long-term growth assays were performed on the A375 wild-type and *PTEN* KO5 and KO11 cells. In the short term, 6-day cell growth assays, the dabrafenib/trametinib combination was able to strongly inhibit growth of wild-type cells, but *PTEN* null K05 and KO11 cells continued to grow at a modest but significant proliferative rate (**Figure 2E**). Growth was also assayed in 1 or 2-to-4-week crystal violet assays where cells were fixed at identical time points (**Figure 2F**) after seeding or were allowed to reach confluence (**Supplementary Figure S4**). The data obtained were consistent, demonstrating that *PTEN* null cells continued to proliferate when compared with *PTEN* wild-type cells in the presence of dabrafenib/trametinib.

### Transcriptomic changes induced by *PTEN* loss

**Figure 3:**
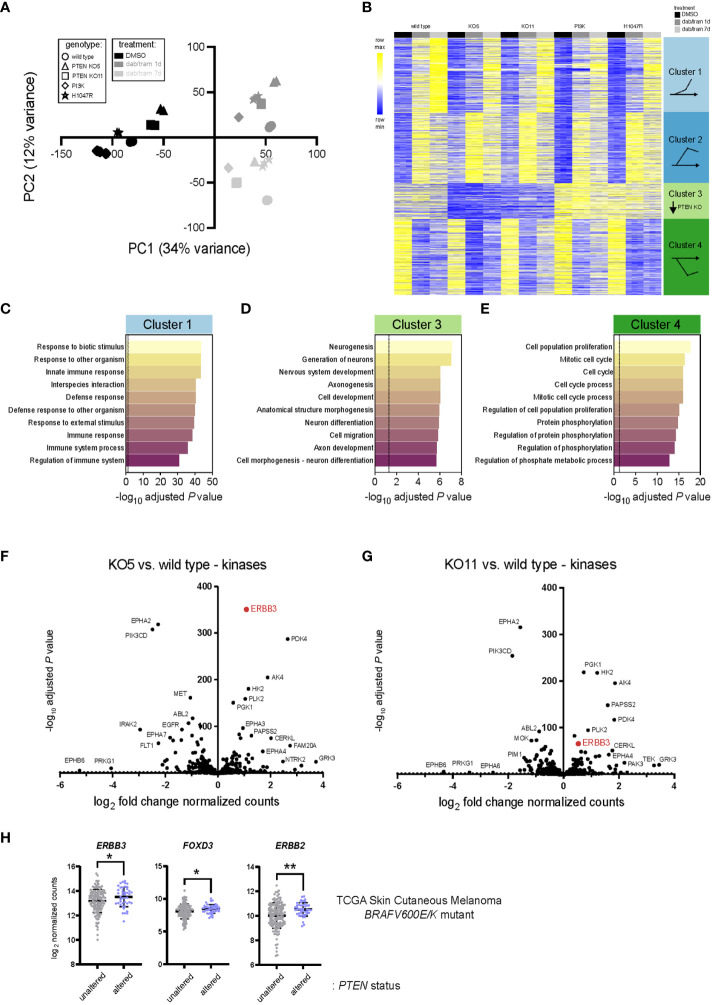
PTEN loss alters the transcriptomic landscape of BRAFV600E melanoma. **(A)** Principal component analysis (PCA) of A375 wild-type, PTEN KO5, PTEN KO11, wild-type PIK3CA (PI3K) overexpressing, and mutant PIK3CA-H1047R (H1047R) overexpressing transcriptomes from three mRNA-seq biological replicates. Component 1 (dab/tram treatment) accounts for the largest possible variance (34%) in the data set. **(B)** K-means clustering (set to 4) of the top 2,000 genes (variable expression) was performed using iDEP0.96. Clusters and gene pattern associations are graphically illustrated at the right. **(C-E)** Pathway enrichment from the clusters shown in **(B)** was performed (GO Biological Process Pathway) using iDEP0.96, and the top 10 most significant pathways are shown for clusters 1, 3, and 4. **(F, G)** DESeq2 was used to identify differentially expressed genes in KO5 **(F)** or KO11 **(G)** cells versus wild-type A375 cells at baseline (DMSO). Kinases were annotated and log2 fold change in DESeq2 normalized counts and the −log10 adjusted P-values used for the volcano plot. **(H)** *ERBB3/HER3*, *FOXD3*, and *ERBB2/HER2* log_2_ read counts from *BRAFV600E/K* mutant, *PTEN* unaltered, or *BRAFV600E/K* mutant, PTEN altered skin cutaneous melanoma patient samples accessed from TCGA database (cbioportal.org). Indicated p-values were calculated in GraphPad Prism using an unpaired, two-sample Student’s t-test. *P < 0.05, **P < 0.01.

We next sought to identify the gene expression changes and potential signatures underlying the modest but significant BRAFi/MEKi resistance observed with *PTEN* loss. RNA sequencing (RNA-seq) was performed in biological triplicate on the *PTEN* null clones (KO5 and KO11), wild-type PI3K (PIK3CA)-expressing, activated PI3K (PIK3CA H1047R)-expressing, and wild-type A375 cells to define changes in the transcriptome with loss of *PTEN* expression and during acute (1 day) and chronic (7 day) exposure to dabrafenib and trametinib in combination. Principal component analysis (PCA) revealed that principal component 1 (PC1, 34% variance) likely reflects exposure to dabrafenib/trametinib (dab/tram) treatment compared with DMSO, whereas principal component 2 (PC2, 12% variance) modestly reflects treatment duration and genotype (*PTEN* or ectopic PI3K status) (**Figure 3A**, **Supplementary Table 1**). Unsupervised hierarchical clustering revealed similar patterns in the expression data (**Supplementary Figure S5A**).

To assign and interpret meaning from the most differentially expressed genes, K-means clustering was performed on the most variable 2,000 genes and those with HGNC-approved official gene symbols were displayed (**Figure 3B**). Default settings of four clusters revealed gene expression patterns that could be defined by genotype and/or drug exposure. Dabrafenib/trametinib exposure led to the upregulation and sustained expression of cluster 2 genes, a gradual increase in cluster 1 gene expression, and rapid and sustained downregulation of cluster 4 genes. In contrast, cluster 3 genes were marked by lower expression in PTEN KO clones, irrespective of drug treatment.

Enrichment analysis of Gene Ontology (GO) Biological Process using iDEP0.96 (22) was performed, and the 10 most significant processes are shown (**Figures 3C–E**, **Supplementary Figure S5**, **Supplementary Table 2**). Genes that were upregulated over time (cluster 1) were associated with response to biotic stimuli, immune response, and defense response (**Figure 3C**). Genes that were more rapidly upregulated in response to dabrafenib/trametinib (cluster 2) were associated with adhesion and ion/cation transport (**Supplementary Figure 5B**). Genes whose downregulation was associated with PTEN status belonged to Go Biological Process pathways related to neurogenesis, neuronal differentiation development, and morphogenesis. These data are in agreement with glioblastoma models where *PTEN* loss induces dramatic transcriptomic changes and a different phenotypic state from the wild type (32). Consistent with growth inhibition observed in all cell lines in response to dabrafenib/trametinib, irrespective of *PTEN* status, cluster 4 genes were associated with cell-cycle progression and mitosis (**Figure 3D**). Cell lines with overexpression of PIK3CA or the active mutant (PIK3CA-H1047R) were notably distinct, possibly due to ectopic expression rather than the use of genome editing of the *PIK3CA* gene. Due to the use of overexpressed PI3K and the higher frequency of *PTEN* alterations observed in patients, we elected to focus further experiments on the *PTEN* KO lines.

To identify potential therapeutic targets in the kinome, we investigated the altered transcripts encoding kinases in A375 cells at baseline (DMSO) compared with the PTEN KO clones. Differentially expressed genes were identified using DESeq2 (21), and kinase genes were annotated using kinome.org (**Figures 3F, G**, **Supplementary Table 3**) (33). Notable significant kinases upregulated at baseline in PTEN KO5 and PTEN KO11 cells included metabolic kinases such as phosphoglycerate kinase 1 (*PGK1*), pyruvate dehydrogenase kinase 4 (*PDK4*), hexokinase 2 (*HK2*), and adenylate kinase 4 (*AK4*). These findings are consistent with a regulatory role for PTEN as a metabolic switch and its loss contributing to the Warburg effect and a shift toward glycolysis (34).

A common upregulated kinase gene in both PTEN KO5 and PTEN KO11 cells at baseline was ERBB3/HER3, which had been previously shown to respond to FOXD3 adaptive upregulation in response to BRAF inhibition (33, 35). We wanted to determine whether *ERBB3/HER3* expression was elevated in human melanoma tumors having mutant or deleted *PTEN* versus melanoma with wild-type *PTEN* at baseline. Analysis of human melanoma tumor transcriptome data (RNA-seq) from The Cancer Genome Atlas (TCGA) indicated that the expression of *ERBB3/HER3*, key transcriptional activator *FOXD3*, and the critical ERBB3/HER3 dimeration partner, *ERBB2/HER2*, were significantly elevated in *BRAFV600E/K* mutant melanoma tumors with *PTEN* alterations (n = 44) versus those with wild-type PTEN (n = 143) (**Figure 3H**). Thus, loss of the tumor-suppressor PTEN is associated with upregulated *ERBB2/HER2*, *ERBB3/HER3*, and *FOXD3* expression in *BRAF* mutant human melanoma.

### Activation of the SOX10-FOXD3-ERBB3/HER3 regulatory axis is enhanced by PTEN loss

**Figure 4:**
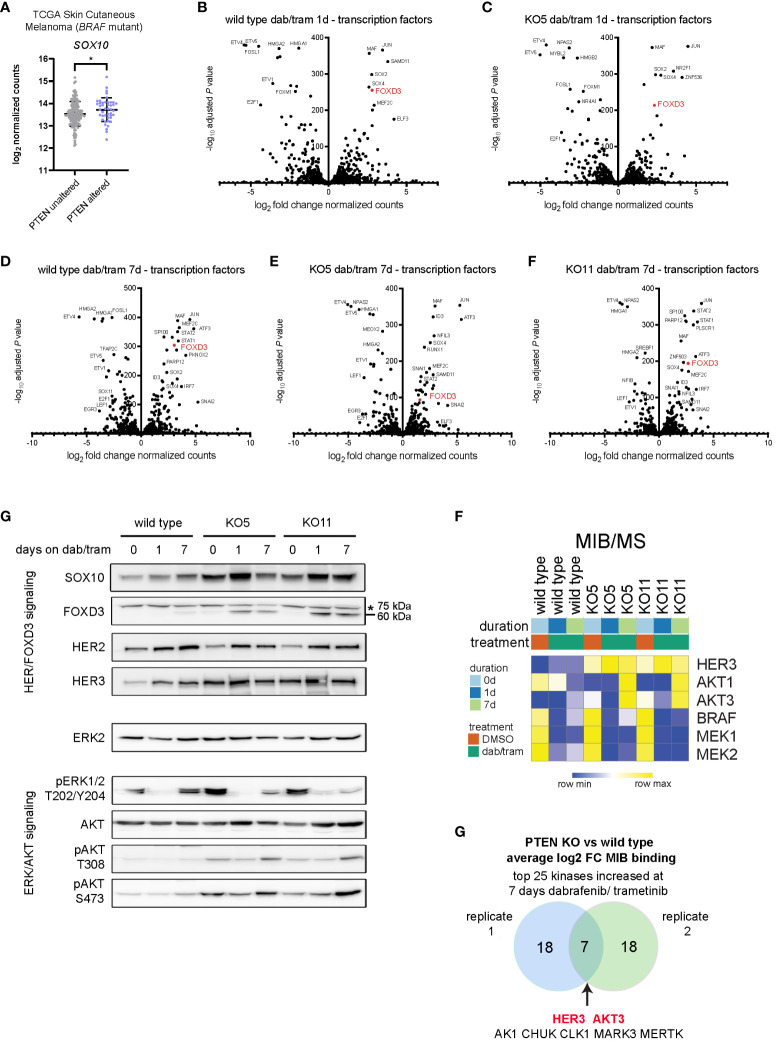
Dabrafenib/trametinib treatment enhances the SOX10-FOXD3-ERBB3/HER3 signaling axis PTEN null melanoma. **(A)** *SOX10* log_2_ read counts from *BRAFV600E/K* mutant, *PTEN* unaltered, or *BRAFV600E/K* mutant, PTEN altered skin cutaneous melanoma patient samples accessed from TCGA database (cbioportal.org). Indicated p-value was calculated in GraphPad Prism using an unpaired, two-sample Student’s t-test. *P < 0.05. **(B-F)** DESeq2 was used to identify differentially expressed genes in wild-type **(B)** or KO5 **(C)** cells, 1 day after 100 nM/10 nM dabrafenib/trametinib treatment, or in wild-type **(D)**, KO5 **(E)**, or KO11 **(F)** cells 7 days after treatment with 100 nM/10 nM dabrafenib treatment versus baseline (DMSO). Transcription factors were annotated and log2 fold change in DESeq2-normalized counts and the −log10 adjusted P-values used for the volcano plot. **(E)** A375 wild-type, KO5, and KO11 cell lines were treated with DMSO (0) or with 100/10 nM dabrafenib/trametinib for 1 or 7 days. Cells were harvested, and immunoblotting for the indicated proteins in the HER/FOXD3 and ERK/AKT axes was performed. FOXD3 has a predicted molecular weight of 60 kDa. A non-specific ~75-kDa band is marked with an asterisk. ERK2 was used as a loading control. Full length blots are presented in **Supplementary Figure S11**. **(F)** A375 wild-type, KO5, and KO11 cells were treated with DMSO (0) or with 100 nM/10 nM dabrafenib/trametinib for 1 or 7 days. Cells were harvested and lysates used for MIB/MS kinome profiling. The heat map displays the normalized MIB binding values (log_2_ label-free quantification (LFQ) intensities) for the indicated kinases. **(G)** Venn diagram of the average log_2_ fold change of MIB binding in *PTEN* KO (clones 5 and 11) vs. wild type for top 25 kinases with increased binding at 7 days of 100/10 nM dabrafenib/trametinib treatment (two biological replicates). The common kinases with increased functional enrichment in *PTEN* null cell lines in response to dabrafenib/trametinib treatment are highlighted in red.

Inhibition of the BRAFV600E-MEK1/2-ERK1/2 pathway in melanoma has been shown by Shao and colleagues to block ERK phosphorylation-dependent SOX10 sumoylation, leading to enhanced SOX10 binding at the *FOXD3* promoter and stimulating the SOX10-FOXD3-ERBB3/HER3 transcriptional axis (36). Analysis of *PTEN*-altered, *BRAFV600E/K* mutant melanoma tumors from TCGA indicated that SOX10 expression was significantly upregulated when compared with BRAFV600E/K mutant, PTEN unaltered tumors (**Figure 4A**). Given the modest but significant capacity of PTEN loss to confer dabrafenib/trametinib resistance, we next examined the effect of drug treatment on the cell line models. Exposure to the BRAFi/MEKi combination of dabrafenib/trametinib for 1 day (1d) led to striking upregulation of *JUN*, *SOX2*, *SOX4*, *MAF*, and *FOXD3* with similar expression changes observed in wild type, PTEN KO5 (**Figures 4B, C**), and PTEN KO11 (**Supplementary Figure S6**). Downregulated genes, consistent with the gene signatures identified in cluster 4 (**Figure 3E**), included the cell cycle regulator *E2F1* and members of the PEA3 subfamily of E26 transformation-specific (ETS) transcription factors—*ETV1*, *ETV4*, and *ETV5*—which are oncogenic transcription factors responsive to MAPK signaling (**Figures 4B, C**) (37). Given that the PTEN KO clones required 7 days or more for observable growth during dabrafenib/trametinib treatment, we next compared the expression of transcription factors at the later time point (**Figures 4D, E**). Surprisingly, the expression of specific transcription factors that were unique to the PTEN KO clones was not readily apparent. FOXD3 expression remained elevated compared with untreated cells of the same genotype after 7 days of treatment, but STAT1/STAT2 signaling (interferon signaling) and ATF3 (stress signaling) were uniquely increased at the longer time point. Interestingly, RNA expression of kinases in the PTEN KO clones after 7 days of treatment suggested increased glycolysis compared with the wild type with increased expression of *AK4* and *HK2* (**Supplementary Figure S6**). PTEN KO clones also both had significantly lower expression of FGFR2 after 7 days of dab/tram treatment when compared with wild-type cells.

Given that the level of FOXD3 transcription at baseline was higher in PTEN KO cells, we next examined the protein levels of factors involved in its function in melanoma cells. Immunoblotting revealed increased protein expression of the SOX10-FOXD3-ERBB3/HER3 axis with inhibition of ERK1/2 activity and increased activation-dependent phosphorylation of AKT at T308/S473 (**Figure 4G**). These data suggested that signaling through the FOXD3-ERBB3/HER3 axis was augmented in PTEN KO clones, but was not driven predominantly by transcription.

To analyze the functional kinome and potentially identify critical targets, we employed our multiplexed kinase inhibitor bead (MIB) enrichment coupled with mass spectrometry (MS) in A375 cells (wild-type and PTEN KO clones) treated for 1 or 7 days with dabrafenib and trametinib. Using type 1 (ATP competitive) kinase inhibitors coupled to sepharose beads enriches for kinases from cell lysates based on activity state, expression, and bead affinity (19, 26, 38). At the protein level, functional MIB/MS kinome profiling showed an increase in ERBB3/HER3 and AKT1/3 binding to the inhibitor beads, whereas BRAF and MEK1/2 exhibited decreased MIB binding, consistent with dabrafenib and trametinib mechanisms of action (**Figure 4F**). Comparing *PTEN* knockout to wild-type cells in independent replicate experiments showed that ERBB3/HER3 and AKT3 were two of the highest bound/captured kinases after chronic (7 day) dabrafenib/trametinib treatment, demonstrating their functional activity in MIB binding resulting from loss of PTEN (**Figure 4G**). Together, these data indicate that BRAFi/MEKi leads to a sustained ERBB/AKT response with its amplitude governed by PTEN status.

### Synergy screens identify a ERBB/HER kinase vulnerability in *PTEN* null A375 cells treated with dabrafenib/trametinib

**Figure 5:**
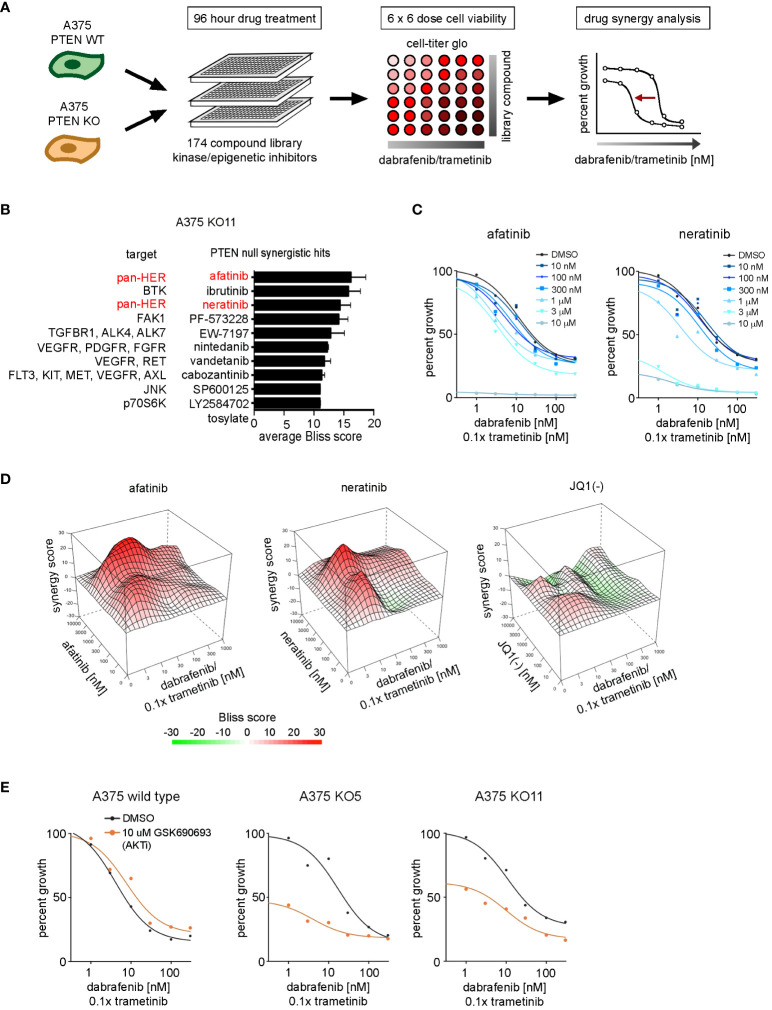
Drug synergy screen against dabrafenib and trametinib in BRAFV600E, PTEN-null melanoma cells. **(A)** Schematic of the synergy screening approach. A375 wild-type cells or PTEN KO clones were plated in 384-well plates and treated 96 h with 6 × 6 concentration matrix of library compound in combination with dabrafenib and trametinib. Cell growth was measured with CellTiter-Glo, and drug synergy was measured using the SynergyFinder package. **(B)** Synergistic compound rankings using Bliss scoring for dabrafenib/trametinib screen in the KO11 cell line. Synergy scores denote the percent inhibition observed following combination treatment, which exceeded the expected growth inhibition as calculated by the bliss independence model. **(C)** 96-h dose–response curves from the complete drug synergy screen show growth inhibition across all doses of the library compound for top synergistic hits, afatinib and neratinib in the KO11 cell line. **(D)** 3D drug interaction landscapes produced using SynergyFinder between dabrafenib/trametinib and neratinib/afatinib or JQ1 (-) in the KO11 cell line following 96-h drug treatment. **(E)** 96-h dose–response curves show synergistic growth inhibition between dabrafenib/trametinib and GSK690693 (AKT inhibitor) only at the 10-μM dose in PTEN null cell lines.

We sought to identify vulnerabilities that could be targeted to reverse the resistance to dabrafenib/trametinib treatment of *PTEN* null cells. A drug synergy screen of a 175-compound, small-molecule library was used to identify inhibitors that could synergize with dabrafenib/trametinib treatment. The compound library, enriched for inhibitors of epigenetic modifiers and protein kinases, including ERBB/HER inhibitors (**Supplementary Table S1**), was used in 6 × 6 dose–response matrices to screen for synergy in combination with dabrafenib and trametinib in the *PTEN* null cell lines (**Figure 5A**). Synergy was measured using the Bliss independence model (Bliss) (**Supplementary Table S5**). Screening identified two irreversible pan-ERBB/HER inhibitors, afatinib and neratinib, as top hits with synergistic Bliss scores in the *PTEN* null cell lines (**Figures 5B, C**). 3D mapping of the synergy landscape of PTEN null cells shows synergistic Bliss scores across all dose combinations of afatinib or neratinib with dabrafenib/trametinib using both KO11 cells (**Figure 5D**) and KO5 cells (**Supplementary Figures S7A, B**). Many compounds including the inactive stereoisomer of the BET bromodomain inhibitor, JQ1(-), did not show synergy with dabrafenib/trametinib (**Figure 5D**).

Afatinib and neratinib are FDA-approved drugs for the treatment of non-small cell lung cancer and HER2+ breast cancer, respectively (39, 40). ERBB2/HER2 is the preferred heterodimerization partner of ERBB3/HER3, transcriptionally upregulated in *PTEN* null cells, and this signaling complex activates PI3K/AKT and MAPK pathways. Additional compounds were identified in our screening data, such as ibrutinib (a Bruton’s tyrosine kinase, or BTK, inhibitor). In addition to irreversibly inhibiting BTK, ibrutinib has been shown to have off-target effects as a pan-ERBB/HER family inhibitor in HER2+ breast cancer (41, 42). Analysis of BTK expression levels from the RNA-seq data set revealed that BTK was not expressed in the KO5 or KO11 cell lines (not shown). Therefore, the synergy associated with ibrutinib in the PTEN null cells is likely due to off-target inhibition of ERBB/HER receptors.

HER2/HER3 heterodimer activation stimulates the ERK1/2 MAPK and PI3K-AKT pathways, AKT inhibitors did not display synergy with dabrafenib/trametinib, most likely due to the high dose (10 μM) required to see significant growth inhibition in combination with increasing doses of dabrafenib/trametinib (**Figure 5E**). The AKT inhibitor GSK690693 inhibited growth by 50% for PTEN null cells at 10 µM, but not wild-type cells at baseline in the absence of dabrafenib/trametinib. A similar difference was observed with the PI3K alpha (PIK3CA) selective inhibitor, alpelisib (BYL719) (**Supplementary Figure S7C**), consistent with enhanced growth dependence on the PI3K-AKT pathway in *PTEN* null cells.

### Neratinib mediates reversal of dabrafenib/trametinib resistance in *PTEN* null melanoma cells

**Figure 6:**
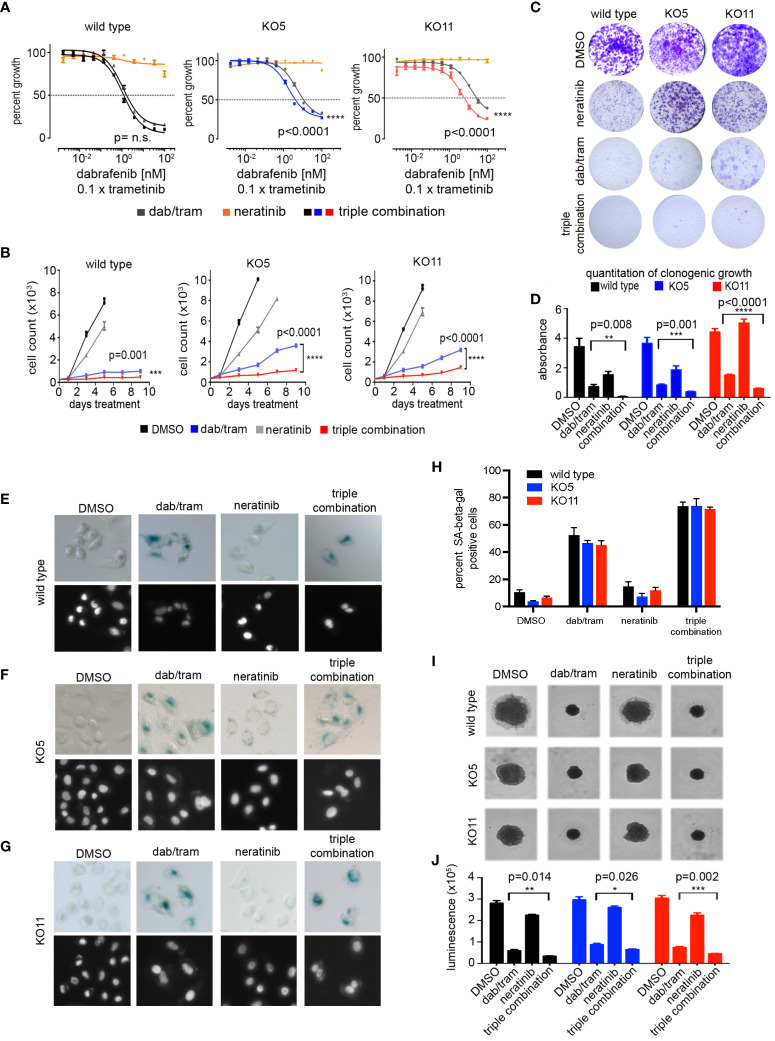
Neratinib synergizes with dabrafenib/trametinib inhibition to reverse resistance in *BRAFV600E*, *PTEN* null cells. **(A)** A375 wild-type, KO5, and KO11 cell lines were treated for 96 h with the indicated dose of dabrafenib/trametinib (dab/tram), 100 nM neratinib, or all three drugs (triple combination) prior to viability measurement using CellTiter-Glo. Dabrafenib (1×) and trametinib (0.1×) were used at a 10:1 dose ratio with or without 100 nM neratinib. Indicated p-values calculated using the extra sum of squares F-test in Prism. **(B)** 9-day cell growth curves across A375 wild-type, KO5, and KO11 cell lines treated with DMSO (control), 10/1 nM dabrafenib/trametinib, 100 nM neratinib, or all three drugs (triple combination). Cells were fixed if confluent before 9 days elapsed. Mean +/− SD, n = 6. Indicated p-values were calculated using two-tailed t-tests. **(C)** Four-week crystal violet assays in wild-type, KO5, and KO11 cell lines. Cells were dosed with DMSO, 10/1 nM dabrafenib/trametinib, 100 nM neratinib, or all three drugs (triple combination). **(D)** Bar plot represents quantification of crystal violet stain in **(c)**, mean +/− SD, n = 3. Indicated p-values were calculated using two-tailed t-tests. **(E-G)** Senescence-associated beta-galactosidase staining in A375 wild-type **(E)**, KO5 **(F)**, and KO11 **(G)** cell lines following 96-h drug treatment. Cells were dosed with DMSO, 10 nM/1 nM dabrafenib/trametinib, 100 nM neratinib, or the triple combination. Nuclei were counter-stained with Hoechst 33342. **(H)** Bar plot represents quantification of percent of beta-galactosidase positive cells treated and stained as in **(E-G)**. At least 200 cells were counted in biological triplicate. **(I, J)** 3D spheroids with A375 wild-type, KO5, and KO11 cell lines treated 7 days with DMSO, 10/1 nM dabrafenib/trametinib, 100 nM neratinib, or all three drugs (triple combination). Brightfield images were taken at 7 days. Cell viability was measured at endpoint with CellTiter-Glo 3D reagent. Mean +/− SD, n = 3. Indicated p-values were calculated using two-tailed t-tests. *P < 0.05, **P < 0.01, ***P < 0.001, ****P < 0.0001

*PTEN* null cells have a diminished sensitivity to growth inhibition with increasing concentrations of dabrafenib/trametinib compared with wild-type A375 cells (**Figures 2D–F**), and ERBB/HER kinase inhibitors were found to reverse the diminished sensitivity (**Figures 5B–D**). In combination with 100 nM neratinib, the IC_50_ for dabrafenib/trametinib shifted from 5.1 nM to 1.9 nM and from 13.6 nM to 4.9 nM in KO5 and KO11 cells, respectively (**Figure 6A**). There was no significant change in IC_50_ for wild-type A375 cells. A similar shift in dabrafenib/trametinib sensitivity with neratinib was observed with SK-MEL-181 cells having shRNA knockdown of PTEN, with no significant shift in IC50 for wild-type SK-MEL-181 cells (**Supplementary Figures S8A, B**). In 9-day growth assays, dabrafenib/trametinib in combination with neratinib inhibited growth of PTEN null cells significantly greater than dabrafenib/trametinib treatment in the absence of neratinib (**Figure 6B**). The triple combination of neratinib with dabrafenib/trametinib also significantly inhibited growth of PTEN null cells in long-term 4 week growth assays (**Figures 6C, D**), demonstrating the ability of ERBB/HER kinase inhibition to durably suppress resistance of PTEN null cells to dabrafenib/trametinib treatment.

In 2D cultures of A375 cells, dabrafenib/trametinib treatment caused a significant induction of senescence-associated beta-galactosidase staining (**Figures 6E–H**). Senescence was greater in the wild-type A375 cells than KO5 or KO11 PTEN null cells treated with dabrafenib/trametinib. Whereas neratinib alone did not induce significant senescence, neratinib in combination with dabrafenib/trametinib significantly increased the number of senescent PTEN null cells. Lastly, we performed 3D spheroid assays to assess the potency of growth inhibition of BRAFi/MEKi with or without neratinib. As shown in **Figures 6I, J**, the triple combination of dabrafenib/trametinib plus neratinib significantly inhibited the growth of both wild-type and PTEN null cells greater than dabrafenib/trametinib alone. Similar to 2D cultures, neratinib alone in 3D cultures had a modest effect on growth of wild-type and PTEN null cells. Collectively, these data demonstrate that FDA-approved inhibitors of the ERBB pathway can suppress BRAFi/MEKi resistance elicited by *PTEN* loss in *BRAF* mutant melanoma.

## Discussion

40% of *BRAFV600E* melanomas harbor *PTEN* loss-of-function mutations that enhance resistance to BRAF/MEK combination inhibitor therapy (6, 14). PTEN is a well-defined tumor suppressor in many cancers, including melanoma, that negatively regulates the activity of the PI3K/AKT pathway (12). While preclinical studies have shown efficacy (43), inhibitors of PI3K and AKT so far have not had significant clinical benefit in treating *PTEN* null cancers, including melanoma. The recent FDA approval of capivasertib in combination with fulvestrant for hormone receptor-positive, ERBB2/HER2-negative breast cancer (and genetic evidence of *PIK3CA*, *AKT1*, or *PTEN* alteration) suggests that this inhibitor should be explored in similarly altered melanoma. Our goal in this study was to define targetable proteins whose expression and/or activity is increased by loss of PTEN and whose inhibition could exploit a vulnerability, allowing the reversal of the resistance to dabrafenib/trametinib treatment seen in *PTEN* null melanoma.

We used CRISPR to delete *PTEN* from A375 human melanoma cells so that our comparison of phenotypes and pharmacological response to therapeutic inhibition of target kinases would be in an isogenic background. *PTEN* null A375 cells have a significantly diminished cell growth inhibition in response to dabrafenib/trametinib treatment, even when ERK1/2 activity remains inhibited. These results suggest that *BRAFV600E*, PTEN-deficient melanoma cells can rapidly undergo an adaptive shift to a BRAF-MEK1/2-ERK1/2-independent growth stimulatory pathway in response to BRAFi/MEKi combinations. Transcriptomic analysis (RNA-seq) of wild-type and *PTEN* null A375 cells showed that ERBB3/HER3 was the most upregulated protein kinase with loss of *PTEN* expression and was confirmed to be functionally activated by MIB/MS kinome profiling. The increased expression was driven by the well-characterized SOX10-FOXD3-ERBB3/HER3 transcriptional axis (33, 36, 44). In contrast, an adaptive resistance response described by Bernards and colleagues has been described, where low SOX10 expression led to a high TGF-beta/EGFR BRAFi-resistant phenotype (45). SOX10 knockout in melanoma cell lines shifted cells to a more invasive, less proliferative phenotype with dramatically reduced ERBB3/HER3 expression (46). Both studies utilized the A375 cell line, underscoring the need to appreciate the heterogeneity of expression and multiple resistance mechanisms that can arise in tissue culture. Using isogenic wild-type and PTEN knockout lines, our data suggest that *PTEN* status could be an important determinant of the adaptive resistance mechanisms adopted by melanoma in response to BRAFi/MEKi.

We additionally observed evidence of a dramatic phenotypic state change at the metabolic level—increased kinase expression suggesting an increase in glycolysis. Hexokinase 2 (HK2) phosphorylates glucose and represents the rate-limiting first step of glycolysis. HK2 has been linked to *p53* and *PTEN*-deficient prostate cancer, where the loss of *PTEN* was shown to lead to amplified HK2 translation (47). The development of selective and potent inhibitors of HK2 is ongoing to target glycolysis dependence in many cancers (48). Here, we focused on methods and approaches that bias toward identifying receptor tyrosine kinases (RTKs), such as MIB/MS proteomics and a drug screen that focused on RTK and TK inhibitors and epigenetic inhibitors. However, ongoing studies should investigate this possibility in melanoma models as a potential avenue of therapy to combat BRAFi/MEKi resistance.

The induction of *ERBB3/HER3* with *PTEN* loss is consistent with analysis of human melanoma RNA expression data from TCGA, where both *ERBB2/HER2* and *ERBB3/HER3* transcripts are elevated in *BRAF* mutant, *PTEN* altered melanoma tumors compared with those with wild-type *PTEN*. The functional significance of *ERBB3/HER3* expression in primary melanomas and metastases has been studied by immunohistochemistry that determined elevated levels of *ERBB3/HER3* expression was a prognostic marker for poor survival (49). However, the analysis did not take into consideration *PTEN* status.

Synergy screens clearly identified the pan-ERBB/HER inhibitors, neratinib and afatinib, as the most effective inhibitors to reverse the shift in IC50 for dabrafenib/trametinib treatment of *PTEN* null melanoma cells. The disruption of feedback due to MAPK pathway inhibition had been described by Rosen and colleagues in the context of single agent BRAFi, where RAF dimers and the activation of numerous receptor tyrosine kinases was observed. The combination of BRAFi PLX4720 and neratinib (to target EGFR/ERBB) was highly effective in a xenograft model (50). Dent et al. recently demonstrated the effectiveness of neratinib in treating a panel of BRAF mutant melanoma patient-derived xenografts as a single agent or in combination with HDACi (51). Given the concept of genome-driven oncology for precision medicine (52), our studies provide impetus for the assessment of ERBB/HER expression in *BRAF* mutant, *PTEN*-deficient human melanoma samples, particularly when resistance arises to BRAFi/MEKi combinations. The mechanistic basis for the augmented FOXD3-ERBB3/HER3 signaling observed in the context of *PTEN* loss requires further study. The amplified AKT activity could directly or indirectly lead to epigenetic regulation or enhanced protein stability that could contribute to and reinforce the adaptive ERBB3/HER3 response to BRAFi/MEKi. Furthermore, given the success of immune checkpoint inhibition in BRAF-mutant melanoma, the impact of ERBB/HER inhibition on the tumor microenvironment should be analyzed. Limitations of our study include the potential bias of the drug library that was screened, the need for increased cell line numbers (ideally those that are *PTEN* wild type), and a need for *in vivo* testing (ideally in an immune-competent setting).

**Figure 7:**
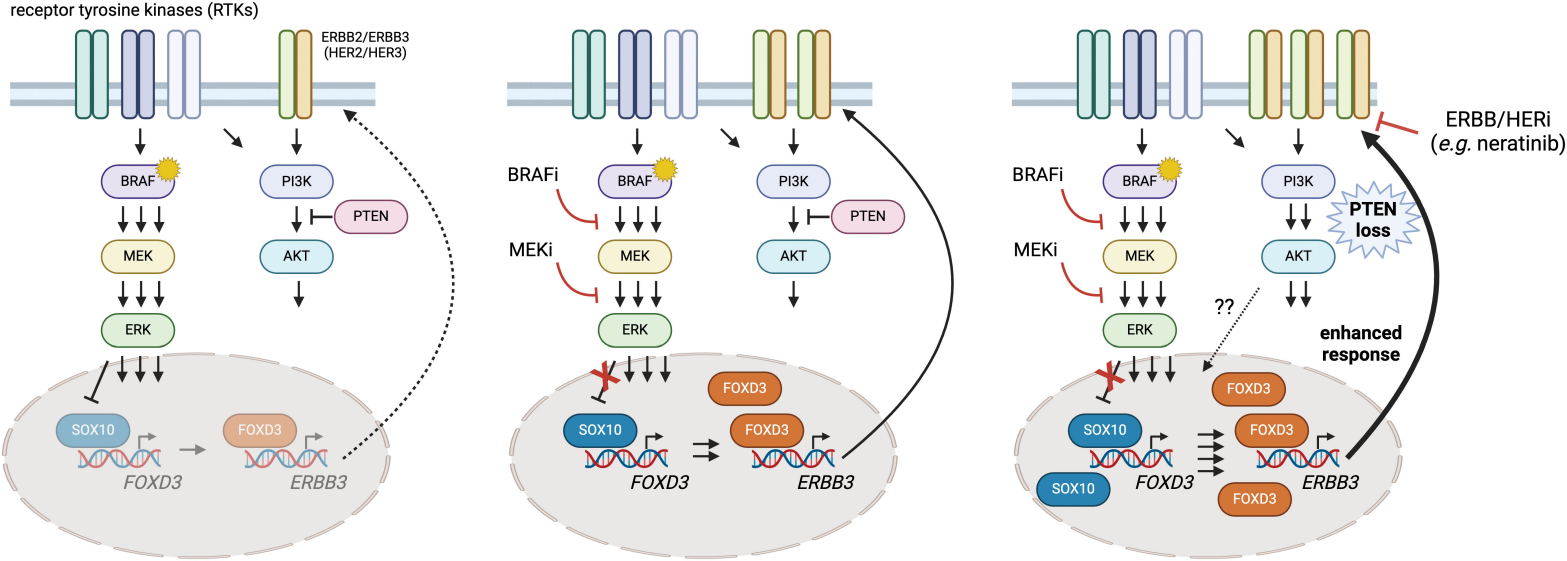
A putative model of heightened SOX10-FOXD3-ERBB3/HER3 signaling in *PTEN*-deficient BRAFV600E melanoma and intervention with neratinib. Melanoma cells exhibit adaptive and intrinsic resistance to targeted BRAF/MEK inhibition that is now the standard of care for BRAFV600E/K melanoma. Previous studies have shown that ERK regulates SOX10, limiting its ability to transactivate *FOXD3* (left panel). BRAFi/MEKi suppresses the MAPK pathway and enables SOX10 and FOXD3 to drive ERBB3/HER3 expression, which can contribute to resistance (center panel). Here, we provide evidence that *PTEN* loss seems to amplify the disrupted feedback leading to hyperactive ERBB3/HER3-AKT signaling. Drug screening identified synergy between BRAFi/MEKi and neratinib (an EGFR/ERBB inhibitor), suggesting that the triple combination of dabrafenib/trametinib/neratinib could suppress the heightened signaling through ERBB3/HER3 and prolong BRAFi/MEKi responsiveness in *PTEN* deficient, BRAFV600E melanoma. Created using BioRender.

Nevertheless, there remains a clinical need for viable strategies where metastatic melanoma patients exhibit intrinsic or acquired resistance to BRAFi/MEKi that is driven by *PTEN* loss. Given that PTEN inactivation also seems to dampen the potential impact of immune checkpoint therapies (18, 53), the identification of additional targets or approaches is a pressing need. Based on our findings, we contend that the strategic use of neratinib with BRAFi/MEKi in ERBB2/HER2-ERBB3/HER3-dependent *BRAFV600E* melanoma with inactivated *PTEN* has the potential to enhance durability of tumor responses and improve patient outcome (**Figure 7**).

## Data availability statement

The RNAseq datasets generated during and/or analyzed during the current study are available in the Gene Expression Omnibus (GEO) (Accession no. GSE255541). The mass spectrometry proteomics data have been deposited to the ProteomeXchange Consortium via the PRIDE partner repository with the dataset identifier PXD033866 and PXD033921. All other data generated and/or analyzed during this study are included in this article (and its **Supplementary Information Files**).

## Ethics statement

Ethical approval was not required for the studies on humans in accordance with the local legislation and institutional requirements because only commercially available established cell lines were used. Ethical approval was not required for the studies on animals in accordance with the local legislation and institutional requirements because only commercially available established cell lines were used.

## Author contributions

ED, GJ, and SA wrote the manuscript. ED, SB, BG, SD, and SA performed the experiments. ED, SB, JB, GJ, and SA contributed to conception, assay development, and experimental design. DM, ED, NS, SR, and SA performed statistical and bioinformatic analysis. All authors contributed to the article and approved the submitted version.
