# Supplementary material for: Neratinib, a pan ERBB/HER inhibitor, restores sensitivity of PTEN-null, BRAFV600E melanoma to BRAF/MEK inhibition
Source: Front Oncol. 2024 May 16;14:1191217. doi: 10.3389/fonc.2024.1191217 (PMC11159048; doi:10.3389/fonc.2024.1191217)
Supplement: Supplementary file 1 [file DataSheet_1.pdf]

SUPPLEMENTAL FIGURE LEGENDS

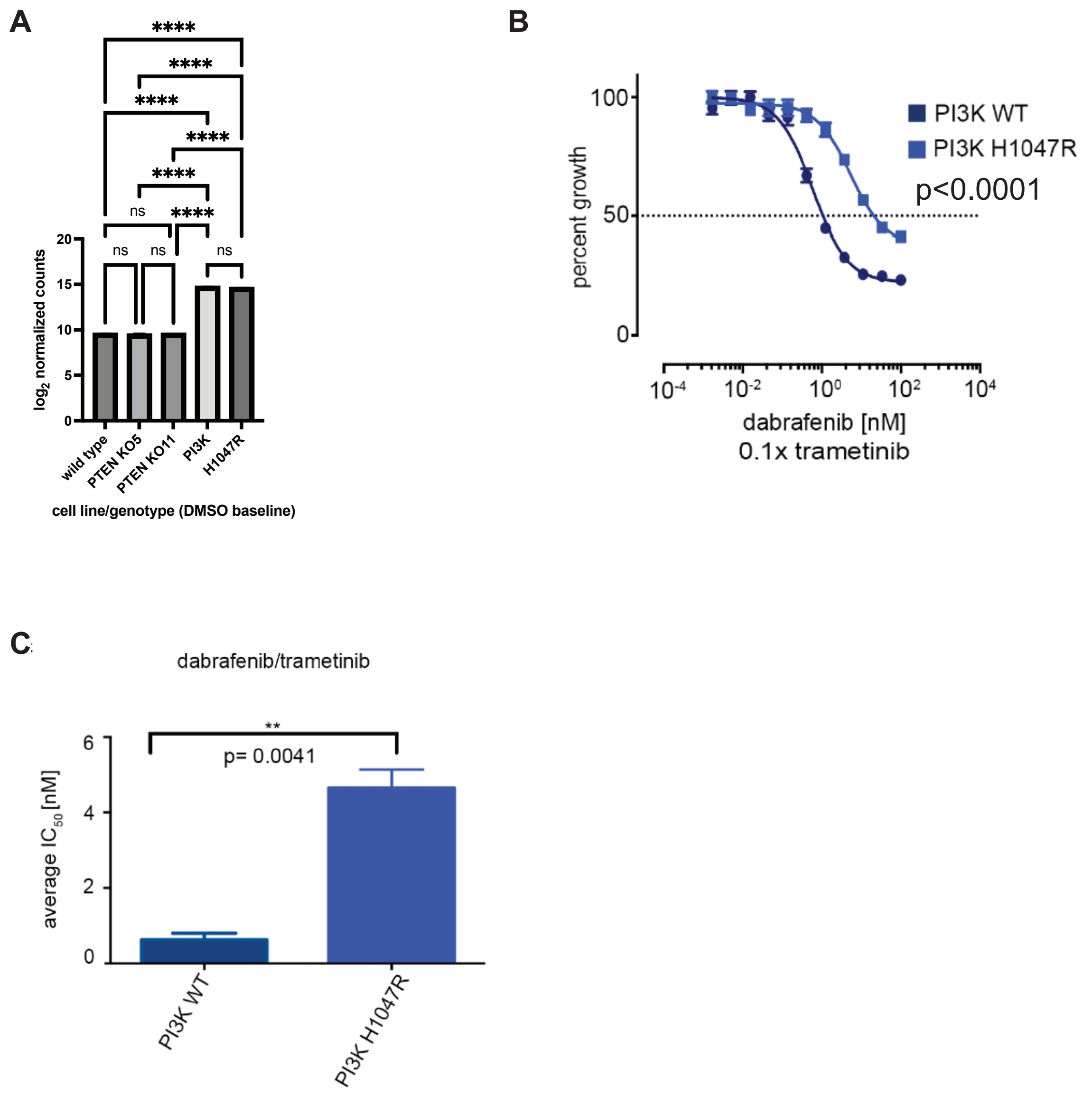

Supplemental Figure S1. PI3K response to combined BRAF and MEK inhibition. (A) Normalized counts for PIK3CA from RNA sequencing shown in Figure 3. A375 wild type, PTEN KO5, PTEN KO11, ectopic wild type PIK3CA (PI3K) or ectopic activated PIK3CA with a H1047R mutation (H1047R) were used for bulk mRNA sequencing. Ectopic PI3K was introduced stably by retrovirus. Log2 transformed, normalized counts (see methods) are shown. **(B)** Polyclonal populations expressing wild type PIK3CA (PI3K) or PIK3CA H1047R were treated with DMSO (control) or the indicated dose of dabrafenib/trametinib for 96 h prior to viability measurement using CellTiterGlo. Dabrafenib (1x) and trametinib (0.1x) were used at 10:1 dose ratio. P-valye was determined using the extra sum of squares F-test in Prism. **(C)** Bar plot indicates average IC50 values calculated by comapring IC50 in the wild type vs. H1047R cell line. P-values were calculated using two-tailed t tests, n=3.

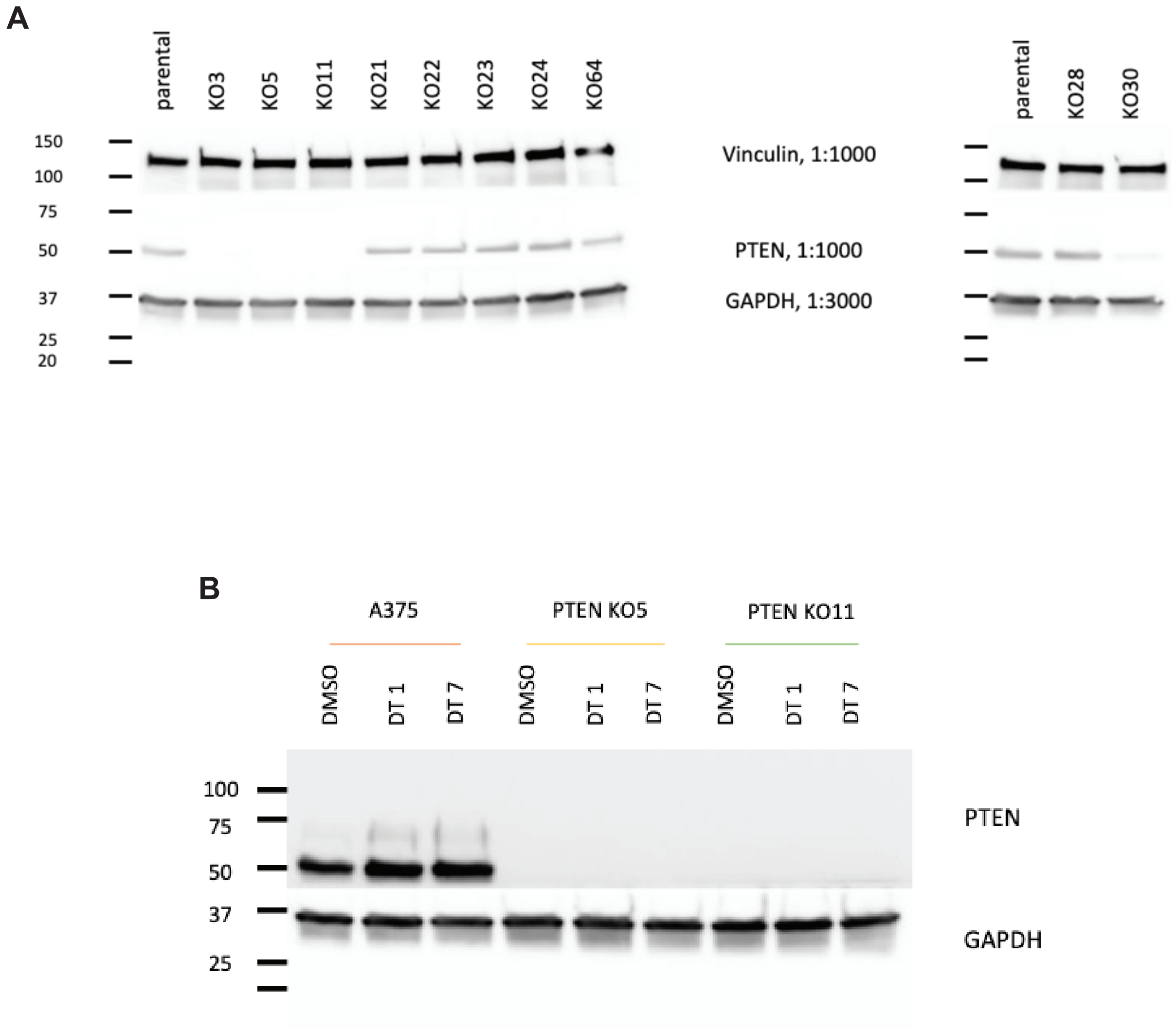

Supplemental Figure S2. Additional A375 PTEN KO clones. (A) A375 cells, transfected with PTEN CRISPR and HDR pools targeting *PTEN* were selected with puromycin and single-cell clones were isolated. Clones KO3, KO5, and KO11 as shown in Figure 2A are included. All other clones had residual PTEN protein detected by immunoblotting. Clone KO30 showed substantial, yet incomplete KO. GAPDH and vinculin were used as loading controls. **(B)** A375, KO clones 5 and 11 treated with DMSO or 100nM/10nM dabrafenib/trametinib for 1 day or 7 days were harvested for immunblotting to detect PTEN. GAPDH was used as a loading control.

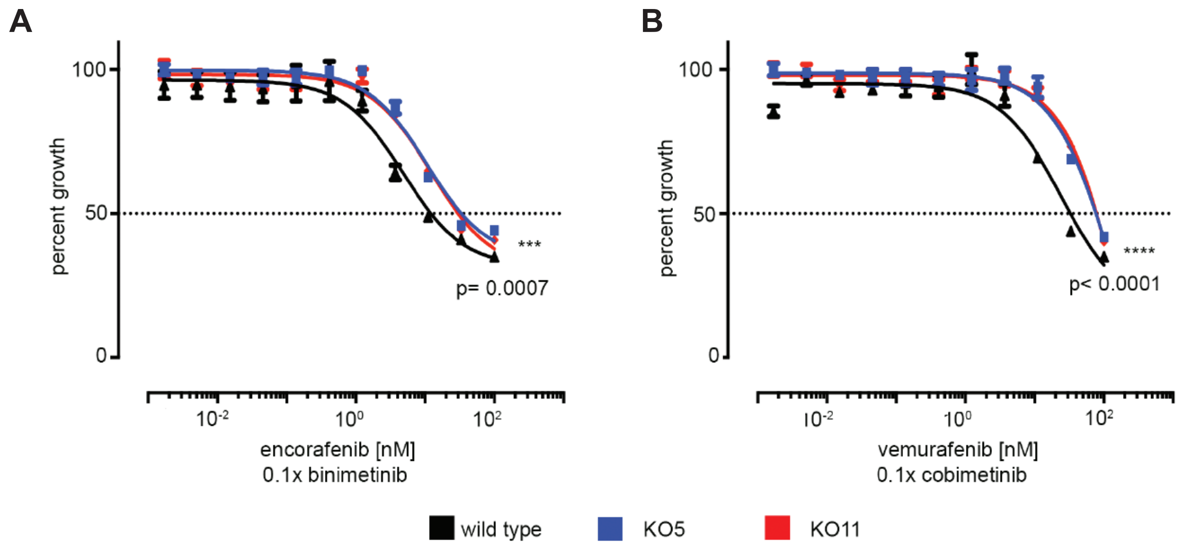

Supplemental Figure S3. BRAFV600E, PTEN null drug response to other FDA approved BRAF/MEK combination therapies. (A-B) A375 wild type and PTEN KO clones 5 and 11 were treated with DMSO (control) or the indicated dose of (A) encorafenib/binimetinib or (B) vemurafenib/cobimetinib for 96 h prior to viability measurement by CellTiterGlo. Encorafenib (1x) and binimetinib (0.1x) and vemurafenib (1x) and cobimetinib (0.1x) were used at 10:1 dose ratio. Indicated P-values were calculated using the extra sum of squares F-test in Prism.

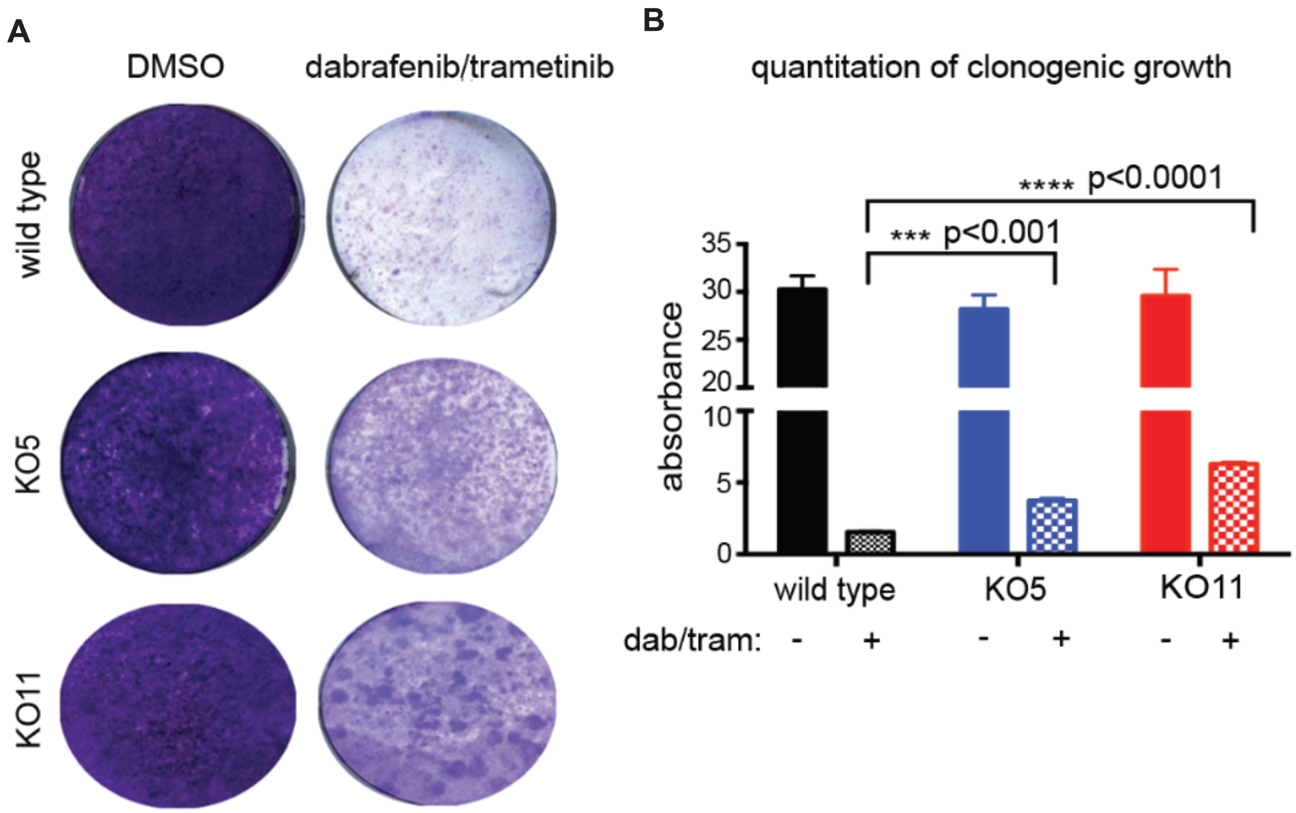

Supplemental Figure S4. PTEN loss confers resistance to BRAFi/MEKi (A) A375 wild type and PTEN KO clones were seeded (2,000 cells per well) in 6-well dishes. The next day, cells were treated with either DMSO or 10nM/1nM dabrafenib/trametinib. Media was replaced every 2-3 days and cells were cultured until the DMSO-treated wells were confluent. Cells were washed in PBS, fixed, and stained with crystal violet. Representative images are shown and **(B)** solubilized quantitation of crystal violet stain is shown. P-vales represent unpaired t-test results in GraphPadPrism.

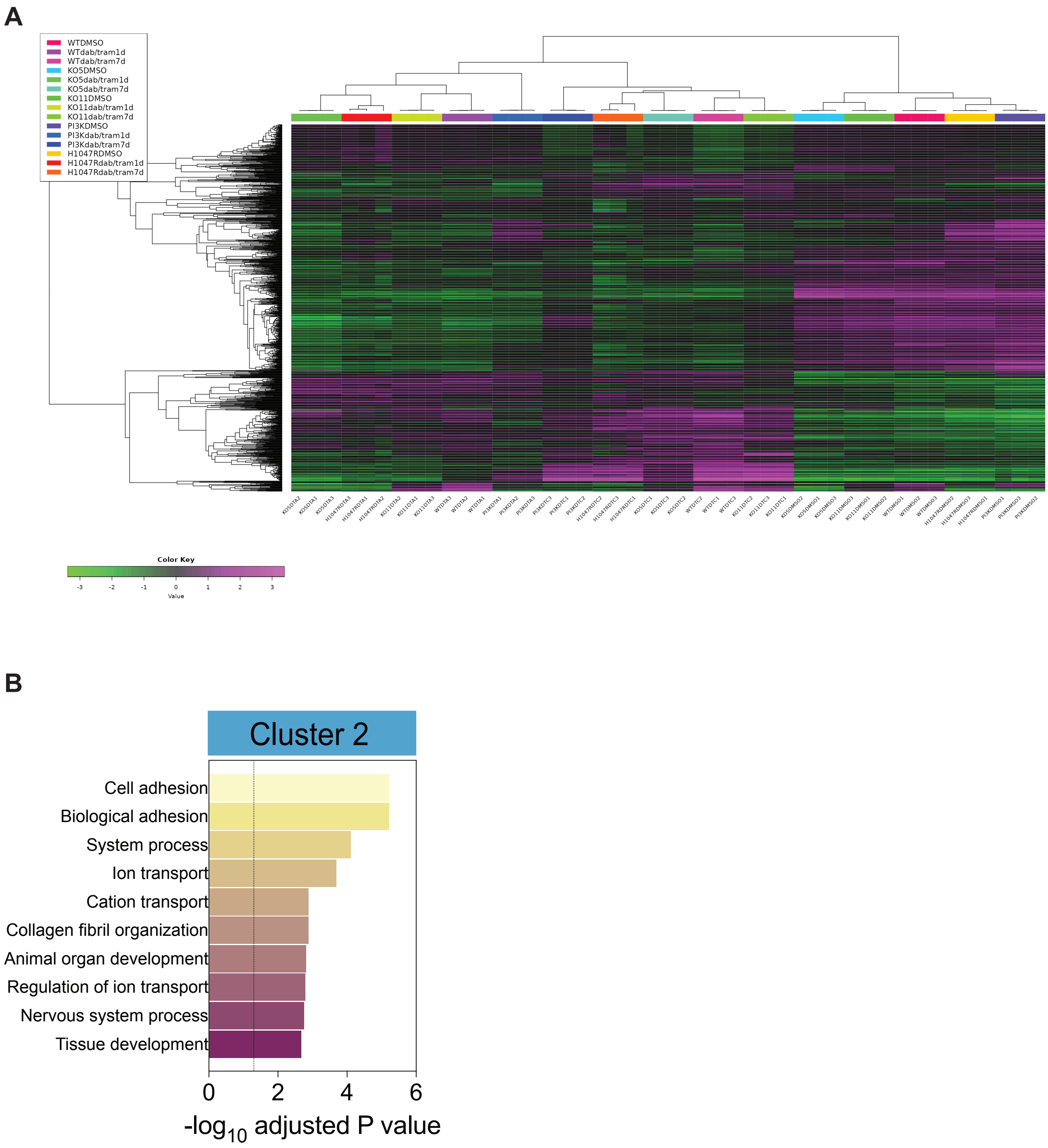

Supplemental Figure S5. RNA sequencing analysis of A375 cells with PTEN KO or PI3K overexpression in response to BRAF/MEK inhibition (A) A375 wild type, PTEN KO5 and KO11 clones or populations stably overexpressing wild type PIK3CA (PI3K) or activated PIK3CA-H1047R (H1047R) were treated with DMSO or with 100nM/10nM dabrafenib/trametinib for 1 or 7 days. Bulk mRNAseq was performed, raw counts normalized using default parameters in iDEP0.96, and unsupervised hierarchical clustering of the top 1000 variable genes was performed (Pearson correlation, average linkage, gene-centered, bar represents Z-score. **(B)** K-means clustering set to 4 clusters. GO Biological Process (top 10) pathway enrichments are shown for Cluster 2 genes (upregulated after 1 d dab/tram and sustained).

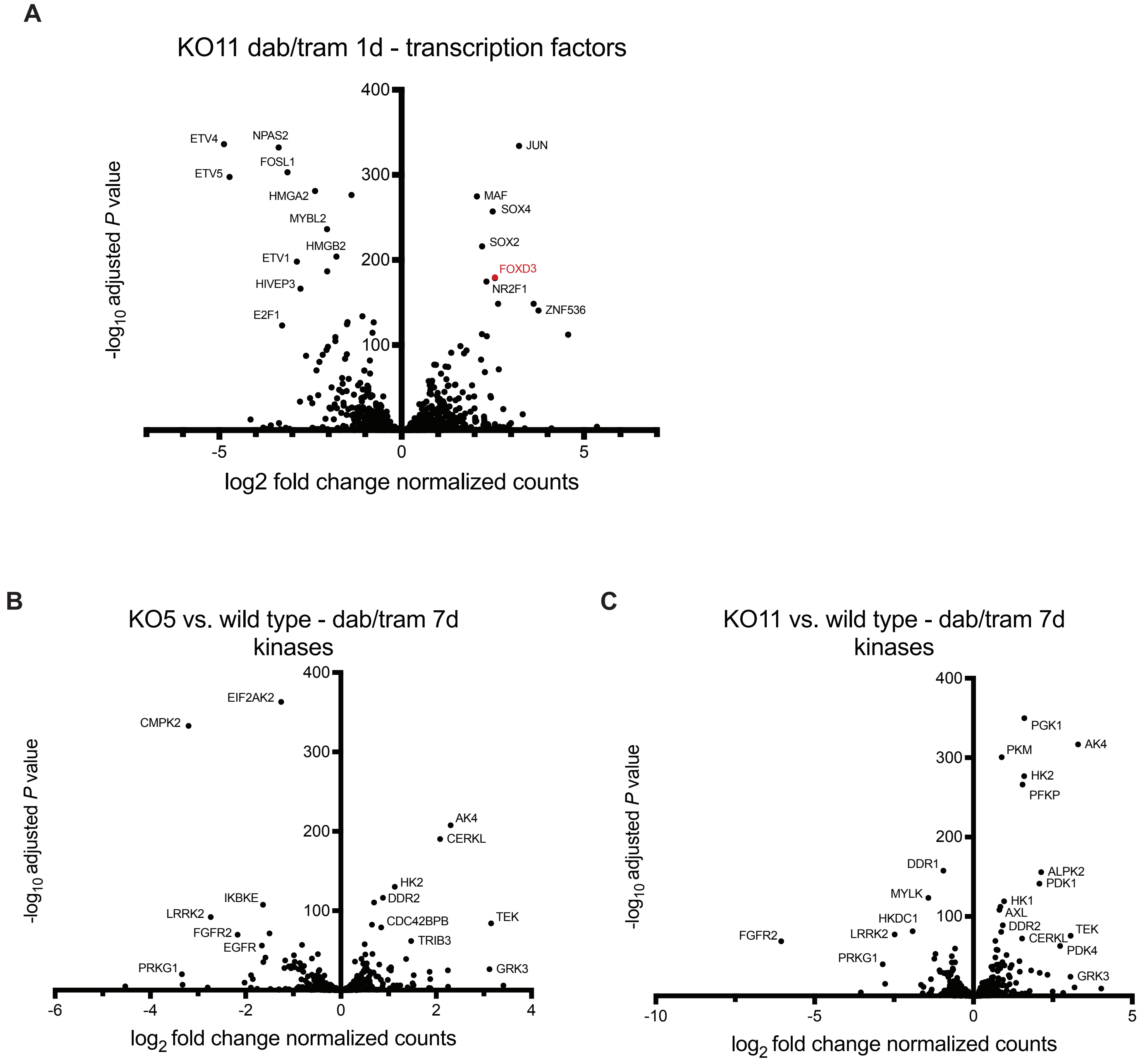

Supplemental Figure S6. Differential expression of transcription factors and kinases in response to BRAFiMEKi and effect of PTEN status (A-B) DESeq2 was used to identify differentially expressed genes in (A) KO11 cells, treated with 100nM/10nM dabrafenib/trametinib compared to DMSO and transcription factors were annotated, or in (B) KO5 or (C) KO11 cells treated with dabrafenib/trametinib (100nM/10nM) for 7 days and compared to similarly treated wild type cells with kinases annotated. The log2 fold change in DESeq2 normalized counts and the -log10 adjusted P-values were used for the vocano plots.

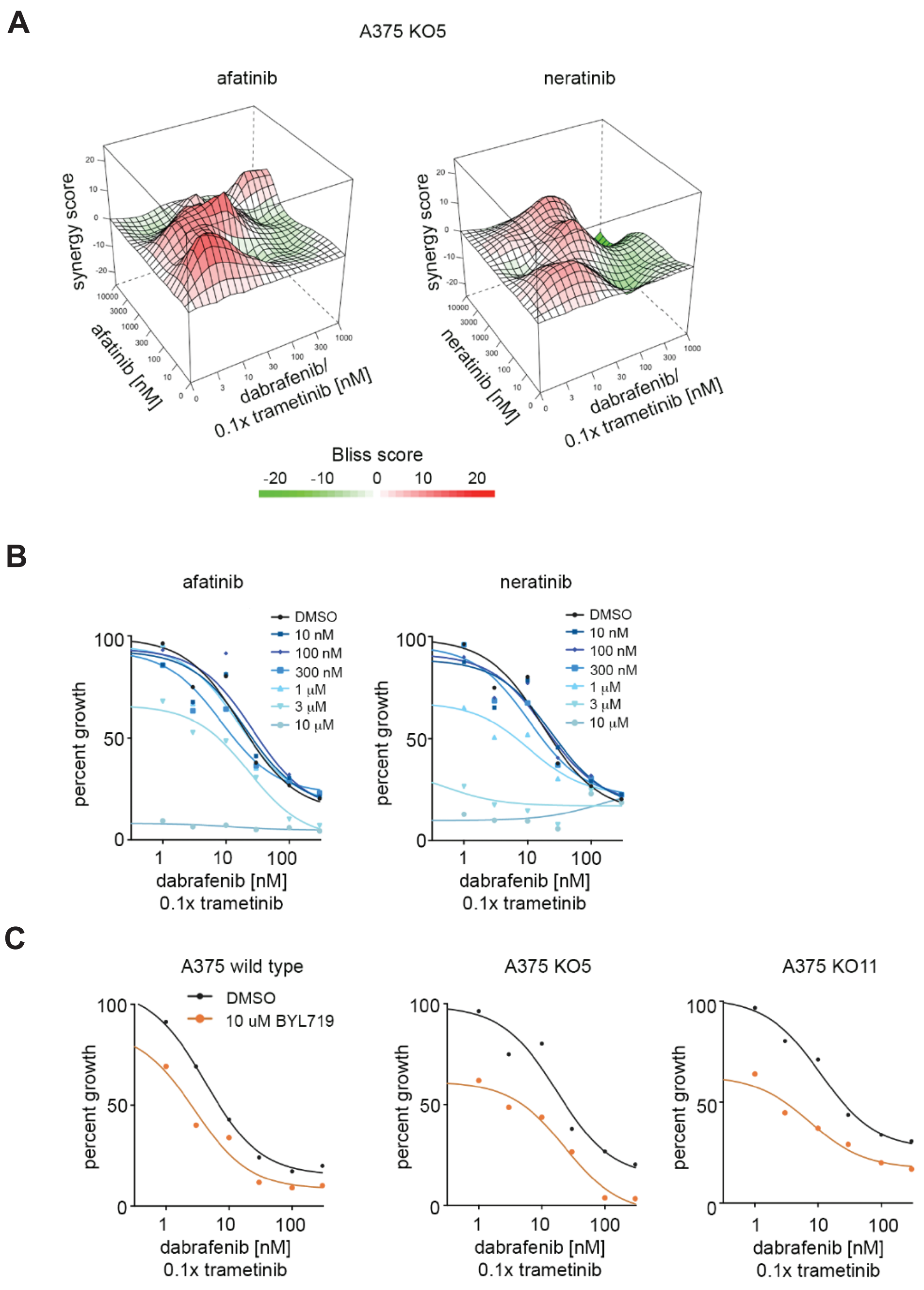

Supplemental Figure S7. Synergy screen results in KO5 cell line.
(A) 3D drug interaction landscapes produced using SynergyFinder between dabrafenib/trametinib and either neratinib or afatinib in the KO5 cell line following 96 h drug treatment. **(B)** 96 h dose response curves from the complete drug synergy screen show growth inhibition across all doses of the library compound for top synergistic hits, afatinib and neratinib in the KO5 cell line. **(C)** 96 h dose response curves show synergistic growth inhibition between dabrafenib/trametinib and BYL719 (PI3Ka inhibitor) only at 10µM dose in PTEN null cell lines.

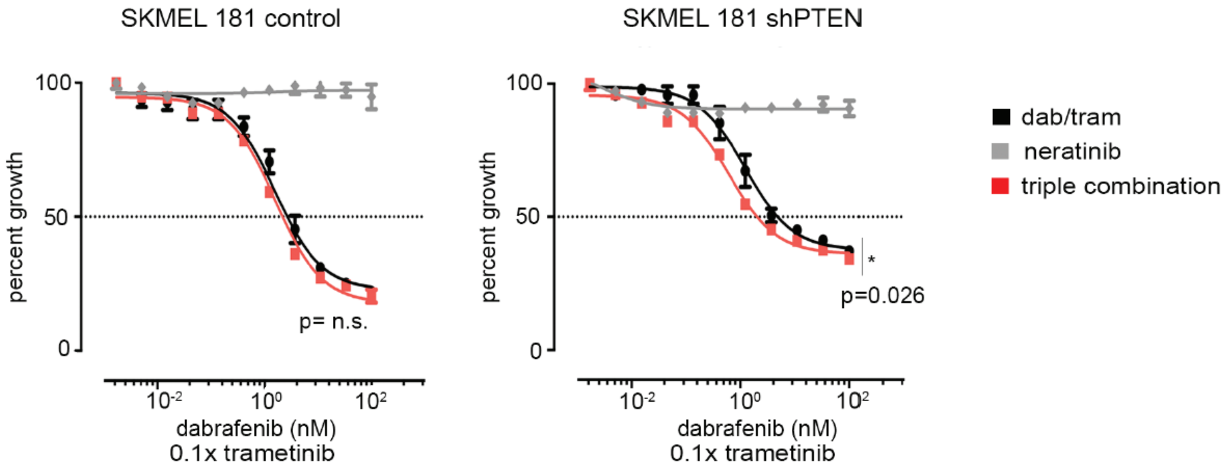

Supplemental Figure S8. Neratinib synergizes with dabrafenib/ trametinib in PTEN knockdown cells to suppress growth.
(A-B) SK-MEL181 cells were infected with lentiviral constructs expressing a non-targeting control shRNA or a PTEN-targeting shRNA. Polyclonal populations of shControl (control) or shPTEN cells were treated for 96 h with the indicated dose of dabrafenib/trametinib, 100 nM neratinib, or all three drugs (triple combination). Cell viability was measured at 96-hours treatment with Cell Titer-Glo. Dabrafenib (1x) and trametinib (0.1x) were used at 10:1 dose ratio with or without 100nM neratinib. P-values were calculated using the extra sum of squares F-test in Prism.

Supplementary Figure S9. Uncropped images of immunoblots in Fig. 1A,B

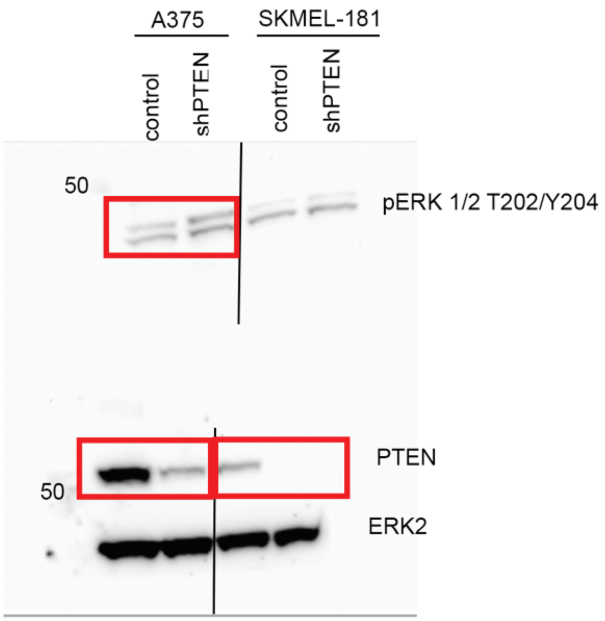

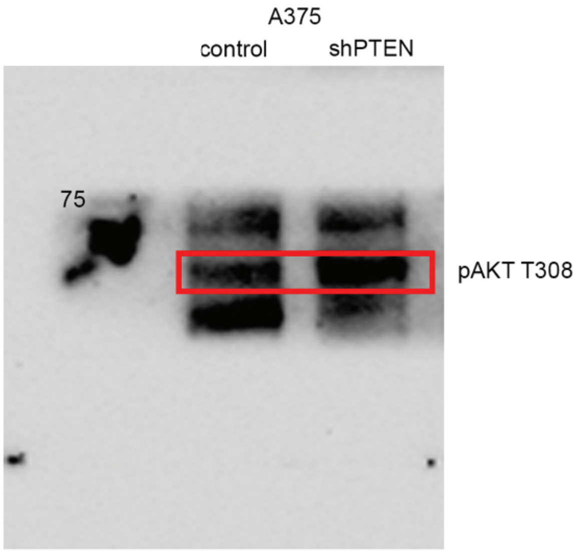

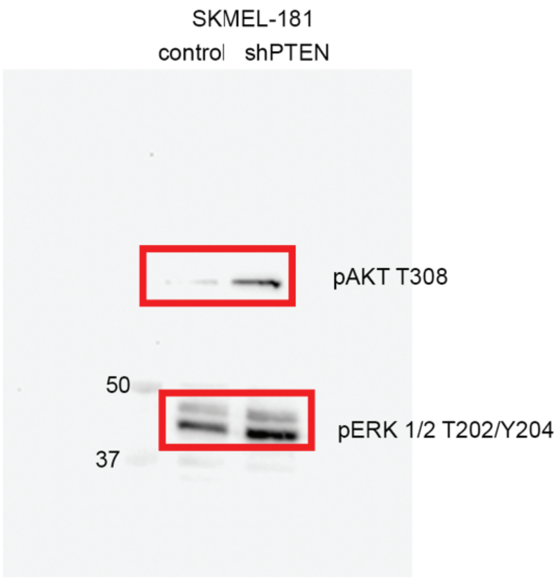

Supplementary Figure S10. Uncropped images of immunoblots in Fig. 2A,B

a

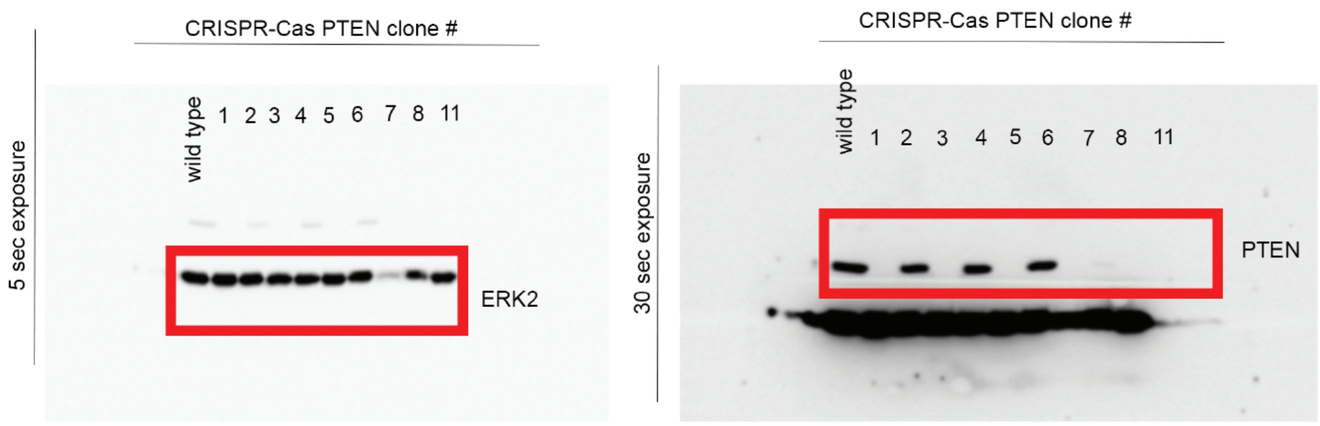

b

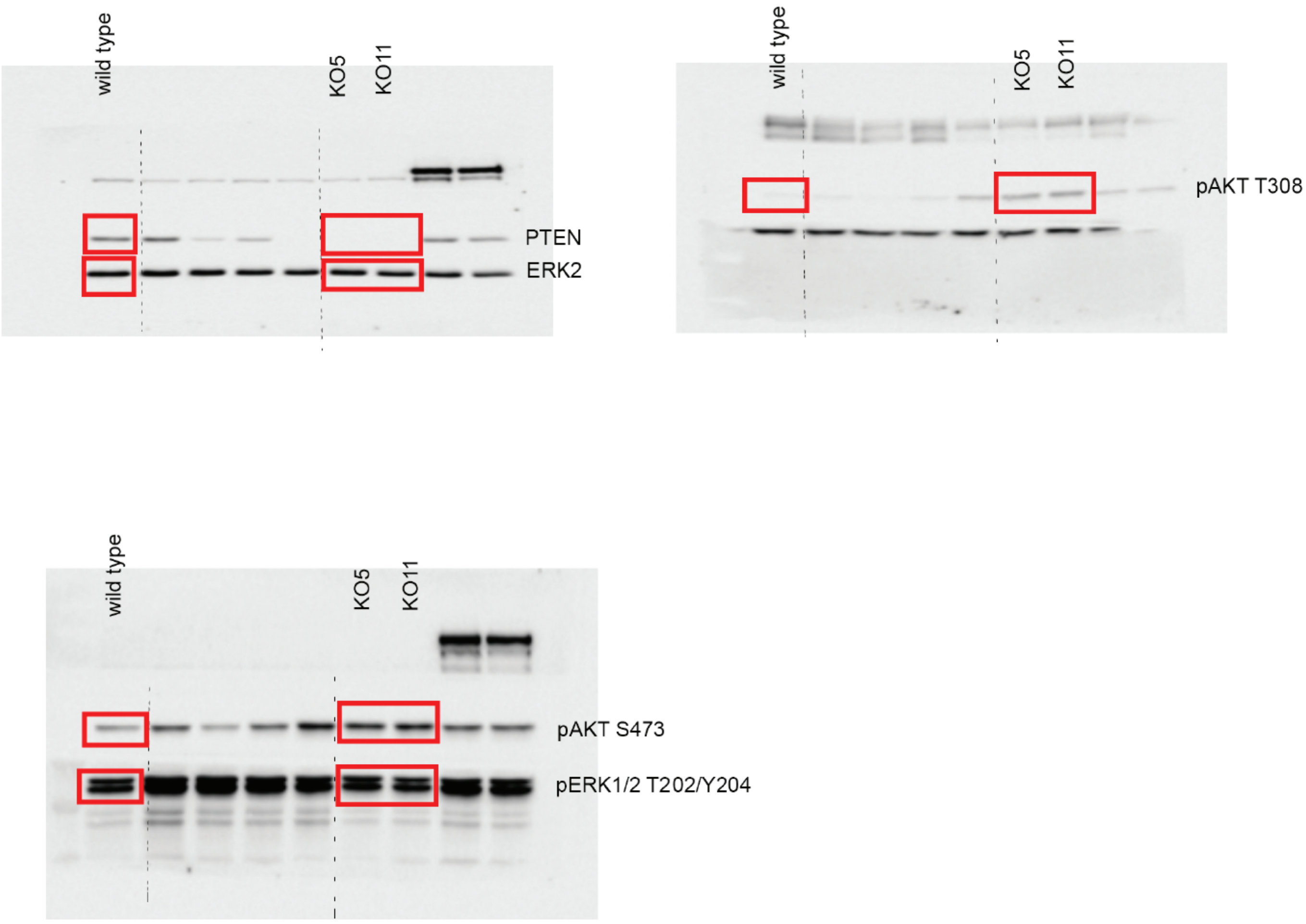

Supplementary Figure S11. Uncropped images of immunoblots in Fig. 4D,E

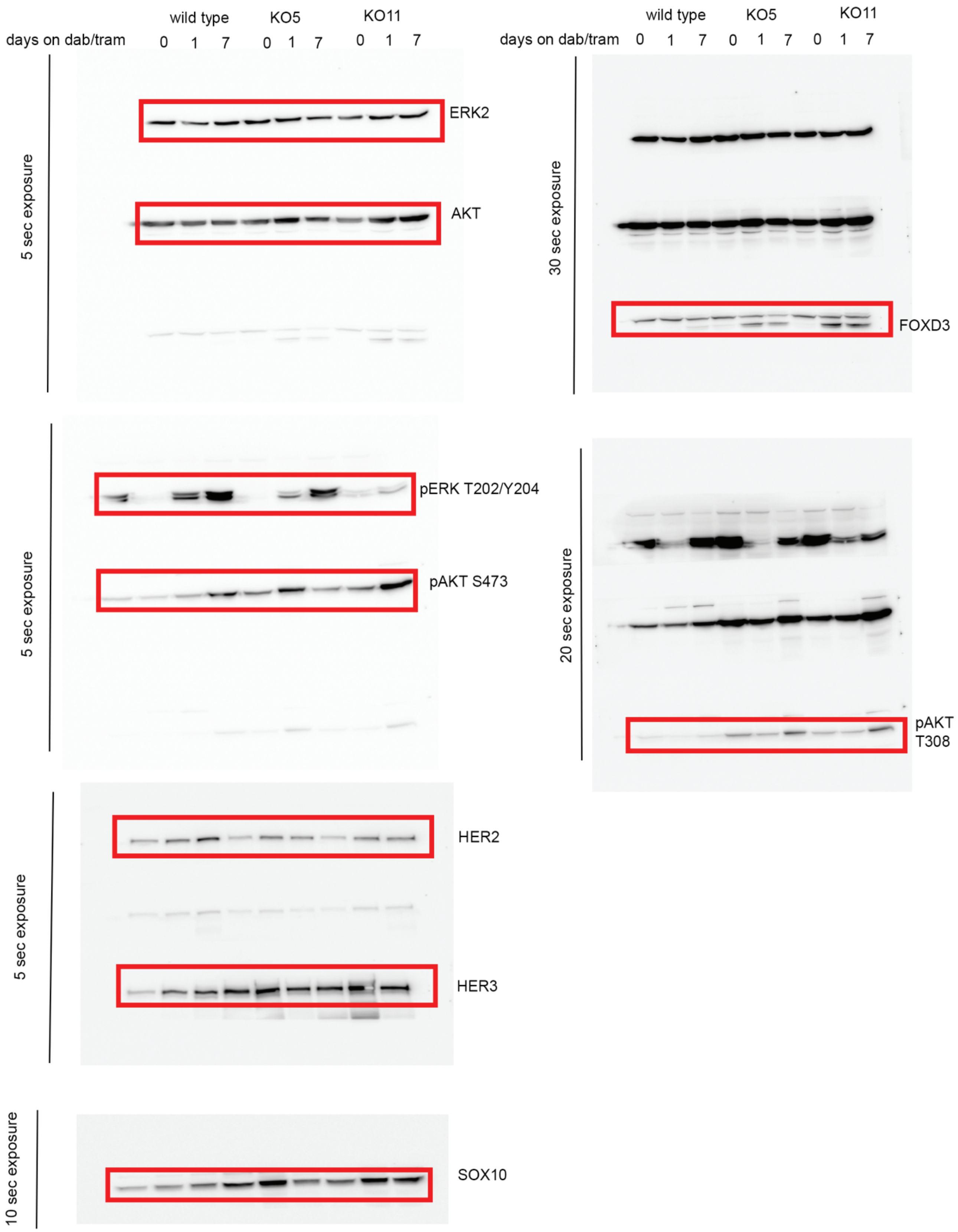

Supplemental Table S1. Processed/normalized RNAseq cpunts used for unsupervised hierarchical clustering and k-means clustering (Excel spreadsheet). DMSO, DTA = dabrafenib/trametinib acute tretament, 1 day), DTC = dabrafenib/trametinib acute tretament, 7 day) KO5 and KO11 (PTEN KO clones), PI3K = wild type PIK3CA overexpressing, H1047R = PIK3CA-H1047R mutant overexpressing. Processed counts from raw count matrix, processed using iDEP0.96 and default settings (see methods).

Supplemental Table S2. K-means clustering and pathway enrichment (GO Biological Process). (Excel spreadsheet). Heatmap values (gene median Z scores) and enrichment (pathway and adjusted P value) from iDEP0.96.

Supplemental Table S3. DESeq2 results. (Excel spreadsheet). Gene, log2 fold change, and -log10 adjusted P value for wild type (WT) PTEN KO5 (KO5), PTEN KO11 (KO11) comparisons - DMSO, DT1 (dabrefinib/trametinib 1 day), and DT7 (dabrafenib/trametinib 7 day).

Supplemental Table S4. Compound list of epigenetic/ kinase small molecule inhibitor library (Excel spreadsheet). Excel file contains information on the vendor from which the library was compounds were obtained, catalog numbers, and the targets of each library compound.

Supplemental Table S5. Synergy scores calculated for dabrafenib/trametinib screens (Excel spreadsheet). Excel file contains Bliss synergy scores generated by SynergyFinder using growth inhibition data from the 6x6 dose dabrafenib/ trametinib screens in A375 wild type, KO5, and KO11 cell lines. The max mean score for the full dose response matrix tested and the max score of the most synergistic dose of each library compound are listed for each cell line. “NA” values represent data for which Synergy Finder failed to produce a score.

Supplemental Table S6. MIB-MS log2 LFQ Intensities (Excel spreadsheet). Excel file contains log2 transformed LFQ intensity values for each captured kinase. Each tab contains an experimental replicate.
